# Bacterial and Fungal Communities in a Degraded Ombrotrophic Peatland Undergoing Natural and Managed Re-Vegetation

**DOI:** 10.1371/journal.pone.0124726

**Published:** 2015-05-13

**Authors:** David R. Elliott, Simon J. M. Caporn, Felix Nwaishi, R. Henrik Nilsson, Robin Sen

**Affiliations:** 1 Division of Biology and Conservation Ecology, Manchester Metropolitan University, Manchester, M1 5GD, United Kingdom; 2 Cold Regions Research Centre, Wilfrid Laurier University, Waterloo, Ontario, 2NL 3C5, Canada; 3 Department of Biological and Environmental Sciences, University of Gothenburg, Box 461, 405 30. Gothenburg, Sweden; NERC Centre for Ecology & Hydrology, UNITED KINGDOM

## Abstract

The UK hosts 15–19% of global upland ombrotrophic (rain fed) peatlands that are estimated to store 3.2 billion tonnes of carbon and represent a critical upland habitat with regard to biodiversity and ecosystem services provision. Net production is dependent on an imbalance between growth of peat-forming *Sphagnum* mosses and microbial decomposition by microorganisms that are limited by cold, acidic, and anaerobic conditions. In the Southern Pennines, land-use change, drainage, and over 200 years of anthropogenic N and heavy metal deposition have contributed to severe peatland degradation manifested as a loss of vegetation leaving bare peat susceptible to erosion and deep gullying. A restoration programme designed to regain peat hydrology, stability and functionality has involved re-vegetation through nurse grass, dwarf shrub and Sphagnum re-introduction. Our aim was to characterise bacterial and fungal communities, via high-throughput rRNA gene sequencing, in the surface acrotelm/mesotelm of degraded bare peat, long-term stable vegetated peat, and natural and managed restorations. Compared to long-term vegetated areas the bare peat microbiome had significantly higher levels of oligotrophic marker phyla (*Acidobacteria*, *Verrucomicrobia*, TM6) and lower *Bacteroidetes* and *Actinobacteria*, together with much higher ligninolytic *Basidiomycota*. Fewer distinct microbial sequences and significantly fewer cultivable microbes were detected in bare peat compared to other areas. Microbial community structure was linked to restoration activity and correlated with soil edaphic variables (e.g. moisture and heavy metals). Although rapid community changes were evident following restoration activity, restored bare peat did not approach a similar microbial community structure to non-eroded areas even after 25 years, which may be related to the stabilisation of historic deposited heavy metals pollution in long-term stable areas. These primary findings are discussed in relation to bare peat oligotrophy, re-vegetation recalcitrance, rhizosphere-microbe-soil interactions, C, N and P cycling, trajectory of restoration, and ecosystem service implications for peatland restoration.

## Introduction

Peatlands are wetland ecosystems which cover four million km^2^ and store a third of terrestrial carbon on a global basis[1, 2]. Underlying geology and prevailing hydrological conditions favour water retention leading to the water table remaining permanently at or near the soil surface, severely restricting aerobic microbial decomposition of animal and plant matter, and leading to the accumulation of peat[3]. Peatland ecosystems are under threat through many processes including industrial peat extraction, agricultural encroachment and climate change[4]. There is thus a strong rationale for the protection and rehabilitation of peatlands for the sake of biological, hydrological and carbon capture related ecosystem services[4, 5]. Despite the importance of peatlands, we know relatively little about their microbial communities which are fundamental to their functioning.

Although globally distributed, peatlands predominantly occur in the arcto-boreal zone of the Northern Hemisphere[6]. The UK hosts 15–19% of global blanket bog, a class of ombrotrophic (rain fed) peatland located in upland areas of northern England, Wales and Scotland that has been designated in the EU and UK Biological Action Plan as a priority habitat[7]. One of the most south-westerly extensions of the European blanket bog is located in the Southern Pennines in northern England between the industrial cities of Manchester and Sheffield. This upland blanket bog (c. 650 km^2^) that developed on upland terrain exposed to a cool oceanic climate[8, 9], has suffered airborne deposition of N, S, and metals since the very beginning of the industrial revolution about 200 years ago[10]. Over 70% of this peatland had been classified as being in a degraded condition with extensive areas devoid of any vegetation including the functionally important peat-forming *Sphagnum* mosses[11]. The exposed bare peat is highly prone to erosion from surface water run-off and as a result is incised with a dendritic network of gullies[12]. Additional factors that have contributed to degradation include unmanaged fire, over-grazing, tourism, and climate change[13]. These degraded blanket bogs are at risk of becoming major sources of atmospheric carbon through erosional losses and aerobic mineralisation of peat resulting from water table draw-down[14, 15].

Large-scale restoration efforts in the Southern Pennines were initiated a decade ago[16], informed by earlier pilot studies including those at our study site at Holme Moss carried out 30 years ago[17]. Interventions included lime and fertiliser application to raise pH from 3.5 to 4.5 and facilitate transient growth of lowland nurse grasses (*Festuca*, *Agrostis* and *Lolium* species) for rapid stabilisation of the bare peat surface, followed by application of seed and heather (*Calluna vulgaris*) brash or planting to establish dwarf shrub cover and, most recently, peat-forming *Sphagnum* moss species. The EU Life programme has supported upland blanket bog restoration through the MoorLIFE programme (2010–2015), with an emphasis on maximising biodiversity and recovery of hydrological function and lost carbon sequestration potential[4].

Soil microbial communities have long been recognised in other ecosystems as below-ground ecosystem engineers involved in C, N, P, S and metal biogeochemical transformation that can also directly influence above-ground plant community structure and productivity [18, 19]. In contrast there is presently limited information on the distribution and function of soil microbes in peatland ecosystems[20, 21]. Peatland restoration impacts on soil bacterial or fungal communities have only been investigated in the context of rehabilitation of peat extraction cutovers in Canada and Scotland. These studies in relatively pristine peatland habitats confirm plant species- or litter quality- specific responsiveness of bacterial and fungal communities to vegetation re-establishment[22–24]. Earlier molecular microbial diversity analyses in peatlands provided a valuable estimate of microbial diversity, including the degraded blanket bogs of the Southern Pennines[25], but lacked the detail and depth of coverage now possible through application of high-throughput DNA sequencing[26, 27].

The lack of knowledge concerning the roles of microbes in peatland restoration is recognised in the restoration literature[7], and the application of microbial ecology in this field is severely limited as a result. Nevertheless, restoration projects are affected by the beneficial and detrimental actions of microbes, which may be directly modulated by restoration-linked interventions through e.g. fertilization, water table manipulation and re-vegetation of bare degraded peat. In this work, we suggest that peatland ecosystem restoration projects should therefore recognise and harness the activities of microbes in terms of, e.g., nitrogen fixation, methanotrophy, and beneficial plant growth promoting and root symbiotic associations in order to strengthen intervention proposals and increase their overall success. For this to happen an improved understanding of peatland microbiology is needed, and recent advances in the field of microbial ecology make this more feasible than ever before.

Our hypothesis is that there is a dynamic interaction between soil microbes, edaphic factors, and vegetation in degraded peatlands, which will be evidenced by differences in the soil microbial community associated with degradation and restoration. We employed high throughput sequencing of environmental DNA to identify both bacterial and fungal community structure in peat, within the intermittently saturated acrotelm/mesotelm[28] and rooting zone in degraded moorland peat, and a variety of successful natural and managed restorations at a single monitoring site in the Southern Pennines[29]. The results support our hypothesis and provide a basis to inform further studies which are needed to understand the functional roles of microbes in peatlands, and the impact of environmental change and management strategy upon their activities. Our data support the precept that restoration success depends partly upon the readiness and response of belowground microbial communities to the restoration activity, and that microbial community structure in peatlands may be diagnostic of future degradation risk or the progression or success of restoration.

## Materials and Methods

### Ethics statement

Permission was granted by the landowner Yorkshire Water and the administrative agency, Natural England, for site access and field experimentation.

### Study site

The study site at Holme Moss (53.54°N, 1.87°W, 490–523 m above sea level) is a designated Site of Special Scientific Interest (SSSI) in the Peak District National Park of Northern England. Low annual mean temperature of c. 7.5°C (1994–2006), prevalence of cloud cover, wind and rainfall (about 3561 mm total precipitation per annum) provide ideal conditions for blanket bog development on gritstone bedrock[10, 29]. Prior to the recent restoration activities beginning in spring 2008 the blanket bog was in a degraded condition with extensive areas of exposed bare peat incised with gullies [11]. Due to degradation-related drainage most of the former blanket bog is presently better described as a heather moorland or upland heath[30].

**Fig 1:**
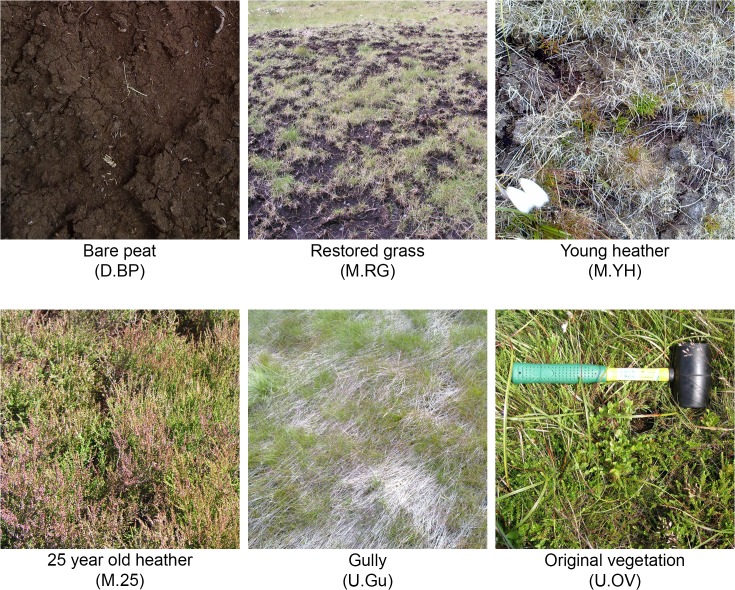
Six vegetation zones investigated in this study at Holme Moss, with identifying name and abbreviation. Further information about each zone is provided in Table 1. Site photographs are provided in S4 and S5 Figs.

In addition to the bare peat we sampled five other distinct vegetation classes that are shown in Fig 1 and described in Table 1. Throughout this paper, these zones are identified with abbreviations that include both the management regime (Degraded, Managed restoration, or Unmanaged natural regeneration) and a description of the vegetation present, as detailed in Table 1.

**Table 1:** Description of the vegetation zones investigated in this study.

Zone	Abbreviation	Description
Bare peat	D.BP	Area devoid of plants characterised by easily eroded bare peat. The sampled areas were excluded from management interventions.
Restored grass	M.RG	Area of recently bare peat semi-restored to grassy area including *Lolium* and *Festuca* species, by application of fertiliser and seed in 2008.
Young heather	M.YH	Newly established heather plants (*Calluna vulgaris*) within bare peat (BP) zones treated with heather brash in 2009.
25-year-old heather	M.25	Heather (*Calluna vulgaris*) dominated site established on land damaged during erection of a radio transmitter mast in 1985.
Gully	U.Gu	Area gullied by water erosion and characterised by water flow or dampness, and exposed bedrock. Characterised by presence of *Eriophorum angustifolium* and acid moorland grasses.
Original vegetation	U.OV	Mature moorland vegetation characterised by diverse flora including crowberry (*Empetrum nigrum*), Cotton grass (*Eriophorum angustifolium*) and bilberry (*Vaccinium myrtilus*).

Each zone is classified as Unmanaged (U), managed (M), or degraded (D), and individually identified by the prominent features of the zone. Photographs of each zone are provided in Fig 1, and site photographs are provided in S4 and S5 Figs.

The impacts of anthropogenic disturbance on the vegetation are evident at the site, which exhibits very low density and diversity of peat-forming *Sphagnum* mosses, and a dominance by sedges such as *Eriophorum vaginatum*. A small portion of the site still supports what we classify as unmanaged original vegetation (U.OV), comprised of *Vaccinium myrtillus* and *Calluna vulgaris* or *Erica tetralix*, similar to the M19 and M20 habitats of the National Vegetation Classification [31]. Management of this moorland area has changed in recent years with the increasing amount of restoration activity. This has necessitated the removal of sheep grazing to enable vegetation re-establishment.

Restoration at Holme Moss was first initiated in 1985 to remediate damage resulting from installation of a large radio transmitter mast, leading to the managed establishment of the restored 25-year-old heather (*Calluna vulgaris*) dominated site (M.25). More recently, fertilizer, lime and nurse grass (M.RG) (including *Festuca*, *Lolium* and *Agrostis* spp.) were applied in April 2008 to the surrounding bare area. Heather brash was then applied in February 2009 to effectively protect and seed the grass areas[16, 32], eventually leading to the emergence of young heather plants (M.YH) within the stabilised grass areas. Some areas of bare peat were deliberately left untreated for this experiment (D.BP). Severe gullying of some bare peat areas has eroded the peat to the gritsone bedrock allowing for natural regeneration of acid grasses albeit in combination with severe loss of peat; we classify these areas as unmanaged gully (U.Gu)[12].

### Sample collection and preparation

Soil cores were extracted from six zones (Table 1) along three parallel c. 300m North-South transects about 80 m apart on 6 July 2010. Along each of the three transects, all zone types were randomly sampled on first occurrence, giving a total of three sampling sites per zone. The 25-year-old restoration (M.25) is in effect a large continuous zone and our 3 samples are effectively from different areas in the same vegetation block. All of the other zone samples are from separate patches in the mosaic arising from degradation and re-vegetation processes. Coring to a depth of 15 cm into the acrotelm/mesotelm and rooting zone was achieved by hammering a plastic pipe (1.2 cm internal dia.) into the peat. Cores for young heather zones (M.YH) were wider in order to accommodate the whole plant. Soil cores were immediately bagged and left within the coring-pipe for transit. In total four cores were taken at each sampling location; three were used for chemical analyses and one was used for cultivation and DNA extractions. The three chemical analysis cores were not treated as independent samples, but were used to obtain reliable mean values. Soil cores for cultivation-based microbiological assays were stored in a dark refrigerator at 4°C prior to analyses the following day. Subsamples of the microbiology cores were stored at -20°C within 12 hours of sampling, and DNA extraction was carried out later using a Powersoil DNA extraction kit according to the manufacturer’s instructions (MoBio Inc., Cambio Ltd., UK). The starting material for DNA extraction was 0.25 g soil at field weight.

### Physico-chemical analyses

Soil samples for conductivity, pH, organic matter and moisture content assays were stored at field weight. Soil moisture content was determined gravimetrically following oven drying at 105°C for 48 h[33]. Organic matter was determined by loss-on-ignition following combustion of 2–5 g of oven-dry peat at 550°C for 4 h[25]. Soil pH and conductivity (TDS Conductivity meter) were measured in a settled slurry of 2.5 g soil in 10 ml deionised water. Ammonium (NH4^+^-N) and nitrate (NO_3_^-^-N) were measured from 5 g air dried peat extracts in 1M KCl following 20 min. orbital shaking at 200 rpm (Stuart SSL1; Bibby Scientific Ltd, UK) and Watman No.3 and 2 μm syringe filtration and dilution (50% v/v) using ion chromatography (Dionex DX 100–275 Dionex Pac CS16 analytical column—IonPacCG16 276 guard column) as previously described[32]. For elemental extraction, 0.5 g air-dried soil was weighed into a 50 ml conical flask and amended with 5 ml concentrated nitric acid (HNO_3_). Following heating at 80°C for 3 h the filtrate (Whatman No. 3) was diluted to 50 ml with deionsed water and subjected to analyses of lead, copper, and zinc in an atomic absorption spectrophotometer (Thermo iCE3300). Phosphorus, potassium and cadmium were determined by ICP-AES (Varian Vista AX—CCD detector).

### Cultivable bacteria and fungi

For enumeration of cultivable bacteria and fungi, 1 g peat samples from each homogenised core were transferred to a 25 ml bottle and extracted by vortexing for 2 minutes in 9 ml sterile distilled water. The supernatant was subjected to repeated 10-fold dilution in further 9 ml sterile distilled water diluents. Dilutions were spread plated on 1/10 Tryptone soy agar medium (pH 7.3) (Difco Microbiology, UK) amended with cycloheximide (50 ppm) (Sigma-Aldrich, UK) and potato dextrose agar medium (pH 5.5) (Difco Microbiology, UK) amended with chloramphenicol (100 ppm) (Sigma-Aldrich, UK) to select for bacterial and fungal growth, respectively[34]. Petri dishes were incubated at 20°C and colonies counted after 48–72 h incubation. Numbers of cultivable bacteria and fungi are expressed as colony forming units (CFU) g^-1^ peat. It should be noted that the cultivation technique used does not accurately represent the environment from which samples were taken, and cultivation techniques in general cannot be expected to enumerate all viable cells in environmental samples[35].

### DNA sequencing of microbial markers from environmental samples

Phylogenetically informative DNA sequences were obtained from each sample by tag-encoded FLX amplicon pyrosequencing targeting the V3 region of the bacterial 16S rRNA gene and the fungal ITS1 region. This analysis was performed by Research and Testing Laboratory (Lubbock, TX), using a Roche 454 FLX instrument with Titanium reagents as previously described[36]. The primers used for bacterial sequencing were 341F (CCTACGGGAGGCAGCAG)[37] and 907R (CCGTCAATTCMTTTGAGTTT)[38]. The primers used for fungal sequencing were ITS1F (CTTGGTCATTTAGAGGAAGTAA)[39] and ITS3R (TCCTCCGCTTATTGATATGC)[40].

### Bioinformatics

Sequence data were processed using UPARSE[41] for quality filtering, denoising, chimera removal, operational taxonomic unit (OTU) clustering, and OTU table generation. Similar to a previous fungal microbiome study[42], we initially performed our analyses using QIIME and found results to be qualitatively similar using UPARSE. We selected UPARSE because it permitted both fungal and bacterial data to be efficiently processed through the same bioinformatics pipeline. The distribution of read lengths and quality scores for both sequencing runs were examined to determine suitable quality control criteria balancing conflicting objectives. The same default UPARSE settings were judged suitable for both bacteria and fungi, including truncation length of 250 and maximum expected error rate of 0.5. These quality control settings were focused on minimising read errors and resulted in approximately half of the reads for both bacteria and fungi being rejected.

For both bacteria and fungi a 97% sequence similarity was used to define OTUs. The Gold database[43] and the UNITE database[44] were used for bacteria and fungi respectively as a reference in the UPARSE pipeline. For bacterial taxonomy assignment the Greengenes database[45] (August 2013 release) was used, and for fungi the UNITE database was used (January 2014 release). Prior to taxonomic assignment of fungi, sequences in the UNITE database lacking phylum level taxonomy were removed. This avoided the problem of >50% of OTUs having no identified phylum when this step was not taken, at the expense of the assignment not necessarily being the closest sequence in the UNITE database. QIIME pipeline scripts[46] were used for taxonomic assignment by BLAST[47].

Identified OTUs were assembled into bacterial and fungal OTU tables summarising the frequency of observation of each OTU in each sample, and these tables formed the basis for determinations and comparisons of community structure. OTU counts were not rarefied to equal sampling depths because this unnecessarily discards data[48]. ITSx[49] (version 1.0.7) and vxtractor[50] (version 2.1) were used to ensure that all sequences used in the analyses possessed the appropriate target regions (fungal ITS1 or V3 region of the bacterial 16S rRNA gene). Data manipulations and statistical analyses were performed using R[51] and the phyloseq package[52] for R. UPARSE commands, OTU clusters, dereplicated quality controlled sequences, taxonomy assignments, R scripts, and other resources sufficient to reproduce the analyses in this manuscript are provided in S1 Protocol. Raw sequence data and metadata are available on the NCBI sequence read archive[53] via study accession SRP048856.

### Statistical Analyses

Measured peat physico-chemical variables and cultivable bacterial and fungal data were compared in pairwise combinations using Mann-Whitney-Wilcoxon tests. P values <0.05 were regarded as significant in all tests.

Each OTU table contained many individual taxa that were subjected to correspondence analysis to summarise and visualise the multidimensional data in a two-dimensional space. Rare OTUs comprising < 0.01% of the sequences detected in the study were excluded from ordinations because they can obscure community patterns[54] and may be differentially detected between samples depending on sequencing depth. Correspondence analysis was performed using the phyloseq wrapper to the Vegan package[55] for R, and used the Bray-Curtis distance measure. We performed unconstrained correspondence analysis to visualise the overall community structure, and constrained correspondence analysis to examine features in the community structure which specifically relate to changes in the above-ground restoration state. Permutation tests (n = 1000) were used to test the significance of measured environmental variables to the microbial community ordinations. Comparisons of microbial communities in bare peat and original vegetation were made using a chi-squared test. Chi-squared P values were corrected to account for multiple comparisons using the false discovery rate method[56].

## Results

### Physico-chemical analyses

**Fig 2:**
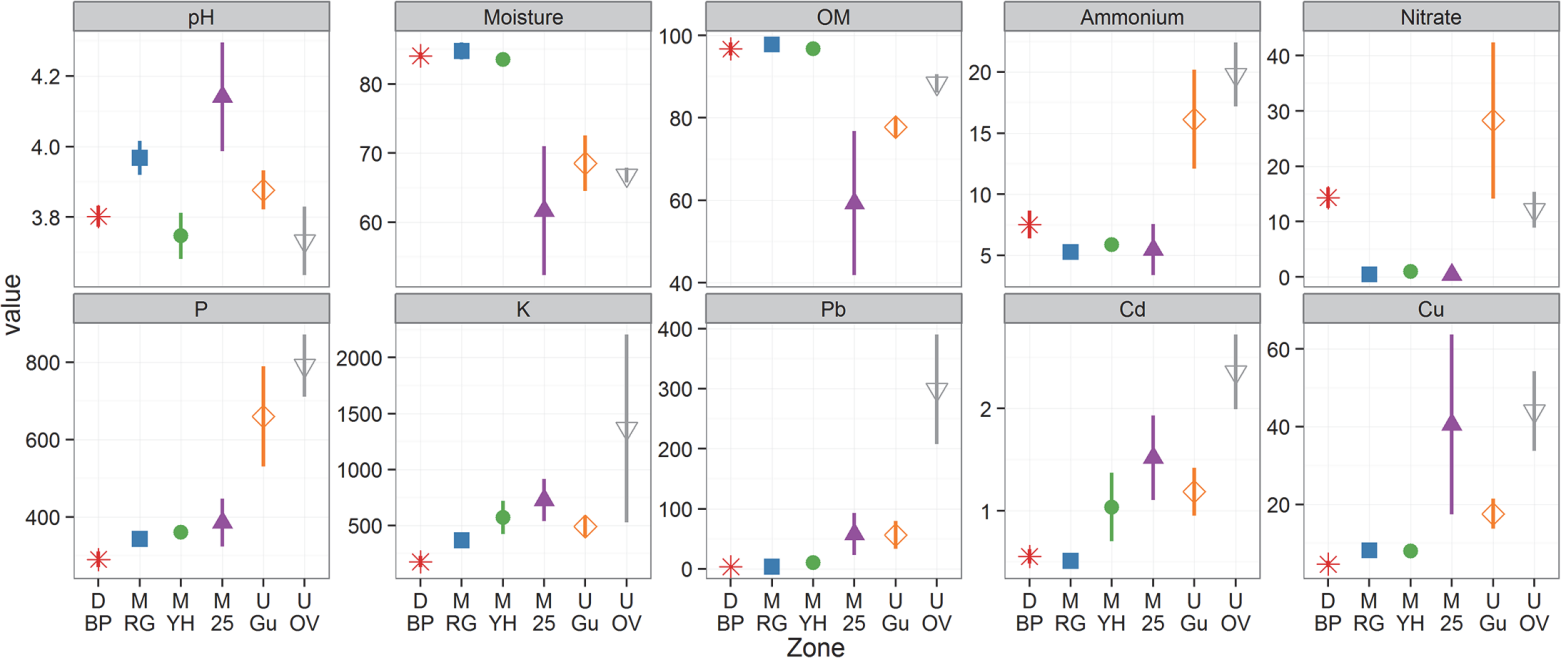
Soil physico-chemical properties in bare peat and vegetated zones (see Table 1) at Holme Moss. All values are expressed in mg kg^-1^ dry soil except for organic matter (OM; %), moisture (%), and pH. Bars indicate the standard error of the mean (n = 3). Different markers and colours are provided to facilitate comparison with other figures. Statistical comparisons are provided in S4 Table.

A summary of the soil physico-chemical data is presented in Fig 2, and full results are given in S1 Table. Statistical comparisons using the Mann-Whitney-Wilcoxon test are summarised in S4 Table. The overall mean pH was 3.9 and no significant restoration-related pH changes were detected. Early stage restorations and bare peat (D.BP, M.RG, M.YH) differed significantly on several measures compared with the longer established zones (M.25, U.Gu, U.OV). Bare peat and the early stage restorations in general exhibited less variation between samples, higher moisture and organic matter (OM), and lower P, and heavy metals. Heavy metals were significantly higher in the original vegetation (U.OV); 11.4-, 2.8-, and 2.5-fold greater than mean values in other zones for Pb, Cd, and Cu respectively. Ammonium and nitrate were significantly higher in unmanaged zones including bare peat (mean 12.7 mg kg^-1^ ammonium, 17.3 mg kg^-1^ nitrate), compared to the managed restoration zones (mean 5.5 mg kg^-1^ ammonium, 0.6 mg kg^-1^ nitrate). Detailed analysis of these results can be found in a Masters thesis[57].

### Microbial enumeration

**Fig 3:**
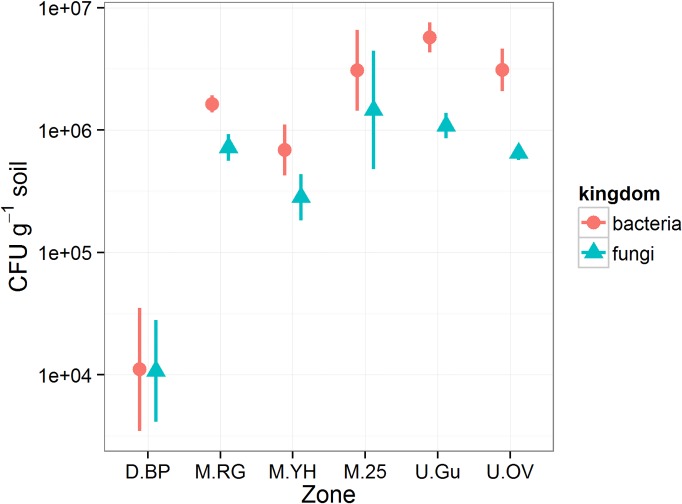
Cultivable bacteria and fungi in bare peat and vegetated zones at Holme Moss. Results are expressed as colony forming units (CFU g^-1^) with bars indicating the standard error of the mean (n = 3). Information about the zones can be found in Table 1 and statistical comparisons are provided in S4 Table.

In the bare peat (D.BP), cultivable bacteria and fungi were both present at approximately 5×10^4^ CFU g^-1^. Cultivable bacteria and fungi in all other sampled zones were over ten-fold higher (p = 0.003 and 0.010; S4 Table), typically around 1×10^6^ CFU g^-1^ (Fig 3). The data indicate organisms that were able to grow under the laboratory conditions tested, therefore the true number of viable cells is likely to be much higher than the counts (CFU g^-1^) presented.

### Microbial community structure

**Fig 4:**
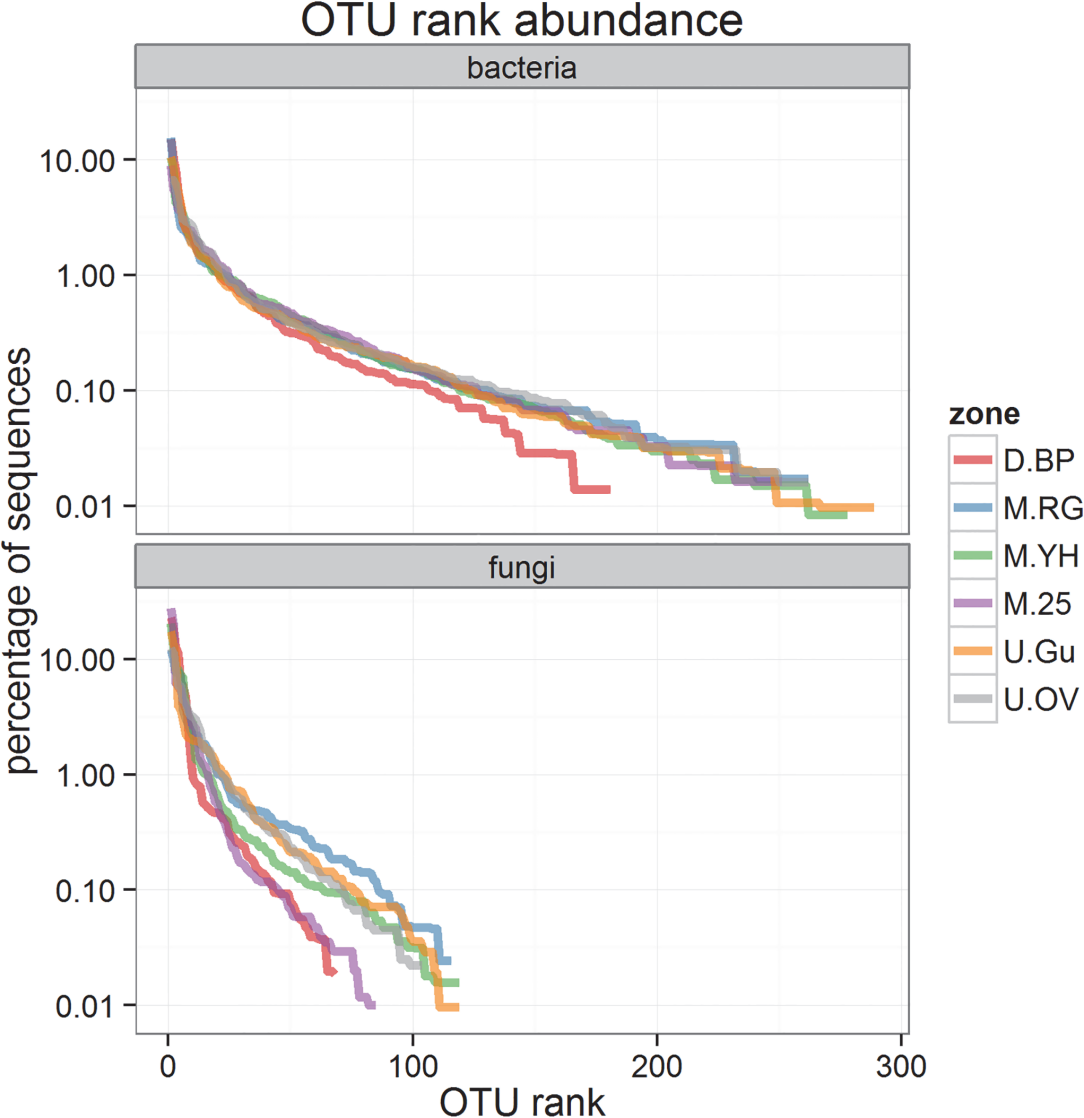
Rank abundance of bacterial and fungal OTUs in bare peat and vegetated zones. Descriptions of the zones are provided in Table 1.

**Fig 5:**
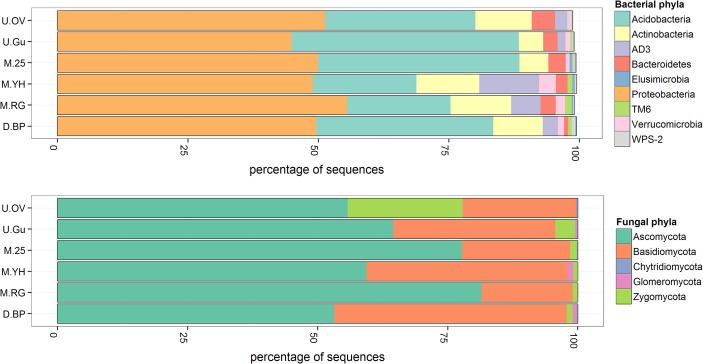
Relative abundance of bacterial and fungal phyla (within each kingdom) in the six zones. The mean of 3 samples for each zone is shown and full results are given in S2 Table. Taxonomic affiliations of all OTUs are provided in S1 Protocol, and descriptions of the zones are given in Table 1.

For each microbial kingdom, approximately 30,000 quality controlled sequences were obtained (see S1 Protocol for full details). These were clustered into 300 fungal OTUs and 441 bacterial OTUs at the 97% similarity threshold, most of which were in the tail of the rank-abundance plots (Fig 4). Fungal sequences were assigned to five phyla which were dominated by Ascomycota (65%) and Basidiomycota (29%) (Fig 5 and S2 Table). Bacterial sequences belonged to 21 phyla of which the most common were *Proteobacteria* (50%), *Acidobacteria* (31%), and *Actinobacteria* (9%) (Fig 5). Further taxonomic information for each OTU is provided in S1 Protocol.

### Microbial community diversity

Rank abundance plots which indicate the relative abundance of each OTU found in each zone (samples combined) are presented in Fig 4, enabling a qualitative assessment of richness and diversity to be made. The steep slope seen in bare peat (bacteria) and 25-year-old heather (bacteria and fungi) indicates dominance of a small number of OTUs, whereas a shallower slope in all other zones suggests a more even population. The richness of each population is indicated by the highest rank on Fig 4. It can be seen that D.BP is host to fewer bacterial and fungal OTUs than all other zones, and that there are less fungal OTUs compared to bacteria. Diversity measures (Chao1, Shannon, Simpson) per zone and per sample (S3 Fig) suggest similar patterns, although the data are insufficient to be conclusive. Indications include lower fungal diversity compared to bacteria, low bacterial diversity in bare peat, and low fungal diversity in bare peat and 25-year-old heather.

### Microbial community relationship to degradation and land management

Most phyla varied in abundance across the vegetation zones, however *Proteobacteria*, the most abundant overall, showed the least variation. Pairwise comparisons between bare peat (D.BP) and original vegetation (U.OV) found significant differences in abundance for most phyla (S3 Table).

**Fig 6:**
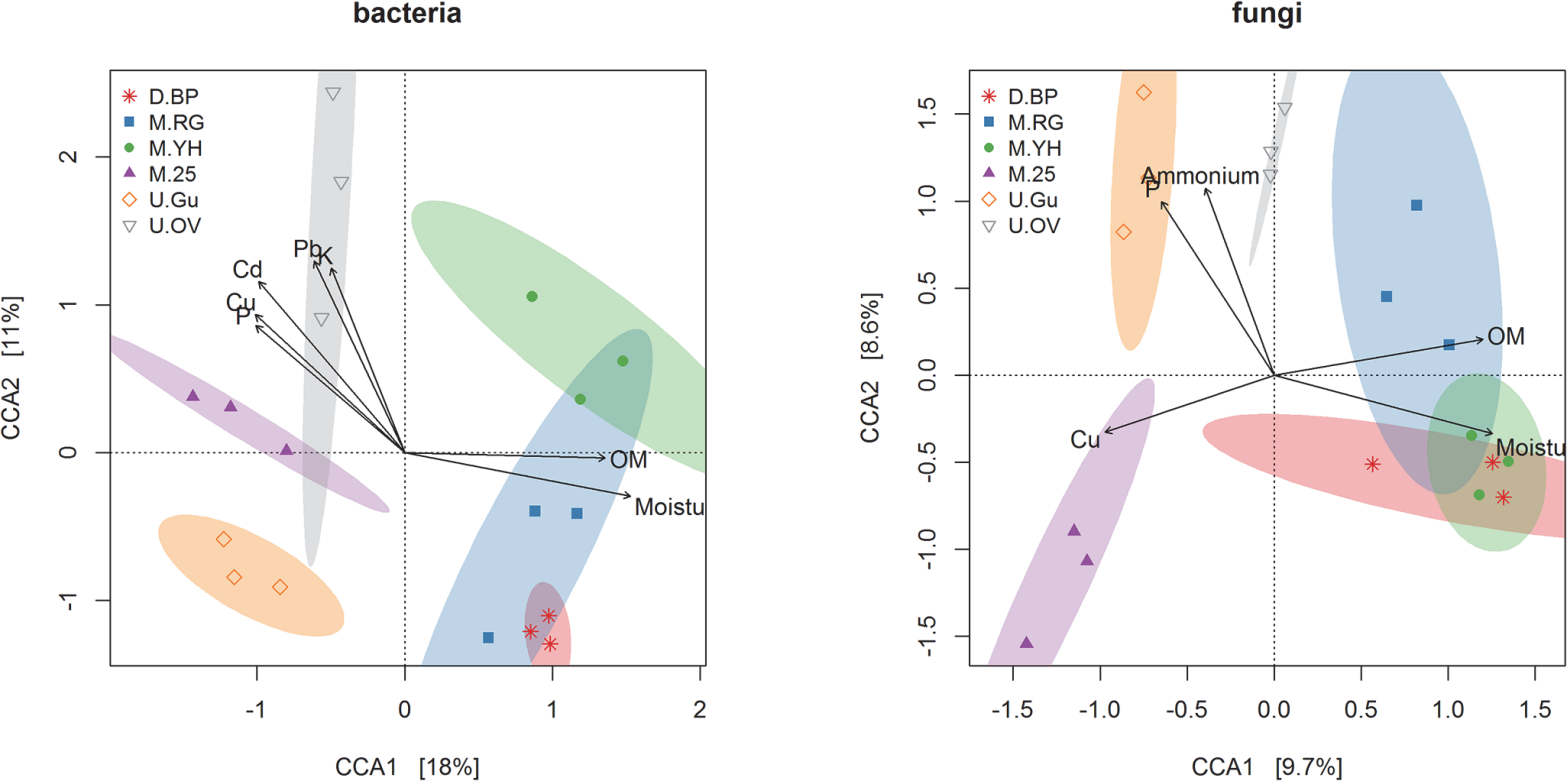
Correspondence analysis of bacterial and fungal communities, constrained by vegetation zone. Analysis is based on relative abundance of 354 bacterial OTUS and 273 fungal OTUs across six zone classifications. Markers indicate individual samples (three per zone type), and dispersion ellipses show the 99% standard deviation confidence interval for each zone. Environmental variables with significance p < 0.05, are shown as biplotted vectors (based on permutation tests; n = 1000). Unconstrained ordinations and scree plots are provided in S1 Fig and S2 Fig.

We used constrained correspondence analysis (CCA) to compare microbial community structure at the OTU level (97% similarity) in each zone, based upon the Bray-Curtis distance measure. This analysis was based on relative abundance of 354 bacterial OTUs and 273 fungal OTUs (rare OTUs < 0.01% were not included). For both bacteria and fungi the belowground microbial community structures are separated on the first two axes of the correspondence analysis (Fig 6). Certain zones form discrete microbial community groupings (e.g. gully for both bacteria and fungi), and some zones cluster together (e.g. the bare peat and restored grass zones for both bacteria and fungi). Unconstrained correspondence analyses and scree plots are provided in S1 Fig and S2 Fig.

Measured environmental variables (Fig 2) that were significant to the correspondence analyses (p < 0.05) are plotted as vectors (Fig 6), showing the direction of increasing value with respect to the ordination axes. Organic matter (OM), moisture, and phosphorus were significant at this level to both bacterial and fungal communities. In addition, many metals were significant to the bacterial ordination and ammonium was significant to the fungal ordination. Moisture and OM increased in the direction of bare peat and early stage restorations for both bacteria and fungi (axis 1 increasing). Potassium and several heavy metals were significant for the bacterial ordination only, increasing in the direction of the original vegetation classification.

## Discussion

Restoration practices intended to halt or reverse degradation of a moorland peat ecosystem were shown to be strongly associated with rapid and concerted changes in surface (acrotelm/mesotelm) soil bacterial and fungal communities. Areas of non-vegetated and eroded peat, that are symptomatic of degraded upland blanket bog[11, 13], support distinct surface soil bacterial and fungal communities compared to peat under adjacent dwarf shrubs communities within the vegetation mosaic. The degradation-related shifts in surface microbial communities are likely to be a contributory factor preventing re-establishment of dwarf shrub vegetation and peat-forming *Sphagnum* species, and observed microbial community associations may provide a much needed below-ground bio-indicator to inform on progress and trajectory of the restoration effort[7, 21].

At the phylum level, bare peat (D.BP) had significantly increased abundance of *Acidobacteria*, *Verrucomicrobia* and TM6, and decreased abundance of *Bacteroidetes* and *Actinobacteria* (Fig 5 and S3 Table) compared to the more elevated non-eroded original vegetation zones (U.OV) supporting a dwarf-shrub community. *Acidobacteria* have been generally recognised as oligotrophs and *Bacteroidetes* as copiotrophs in a meta-analysis[58], which would suggest that the acrotelm of bare peat is more oligotrophic compared to corresponding original vegetation at Holme Moss from a microbial perspective. The increased abundance of Basidiomycota in the eroded bare peat acrotelm, that includes, for example, ligninolytic and cellulolytic members of the Agaricomycetes, further indicate an oligotrophic habitat in surface bare peat. Our chemical data highlight P and K being particularly depleted in bare peat compared to all other zones (Fig 2). Despite the oligotrophic phylum signature in bare peat, OM was relatively high which would normally be expected to favour copiotrophs, however the OM in peat is typically recalcitrant due to anoxic and acidic conditions. In our bare peat areas which are relatively dry and not expected to be anoxic, microbial carbon mineralisation is probably limited instead by the availability of P and K, and possibly other nutrients such as base cations.

A recent survey of the bacterial composition of *Sphagnum* dominated peat wetlands in surface and subsurface layers detected vertical stratification which was attributed partly to differing oxygen requirements[59]. That study focused on poorly studied phyla and candidate divisions including *Acidobacteria*, *Verrucomicrobia*, and candidate division TM6. We detected all of these at increased levels in bare peat compared to the original vegetation, and similarly we also found *Acidobacteria* to be the numerically dominant bacterial phylum in peat. The results of the present study relate only to the top 15 cm of peat which was sampled and mixed, therefore any differences in community structure throughout that depth are integrated and cannot be detected.

Reduced cultivable bacteria and fungi in degraded bare peat compared to any of the vegetated zones (Fig 3) reflect similar previous findings after vacuum extraction of peat[60]. Total direct counts of bacteria in peat are typically in the range 10^8^–10^9^ g^-1^ peat[61], approximately 100-fold higher than our cultivable counts. This highlights the fact that microbes in environmental samples are not easily cultivated on rich general purpose laboratory media, however we expect that the cultivable fraction is stable enough to allow comparison of cultivable microbes between zones. The abundant availability of photosynthesis-derived primary carbon sources e.g., mono- and di- saccharides, amino acids and carboxylic acids through rhizodeposition into the acrotelm/mesotelm further supports a more copiotrophic niche [62] in the vegetated zones that explains enumeration of more cultivable bacteria and fungi irrespective of the vegetation class investigated (Fig 3). The lack of P and rhizodeposition-derived carbon sources may explain a reduced capacity for biologically driven carbon and nitrogen transformations in the bare peat. Reduced *in-situ* carbon loss through microbial respiration previously observed in bare peat at Holme Moss[32], supports our contention that plant establishment is being hampered by reduced bio-available nitrogen. It should be noted however that bare peat offers many additional routes for carbon loss due to its exposure and erodibility[12].

Compared to the other zones, bare peat and early restoration vegetation (M.RG and M.YH) subjected to NPK fertilization and lime[32] show comparable physico-chemical properties except for elevated soil nitrate levels in bare peat (Fig 2). Nevertheless, large areas of bare peat resist re-colonisation by plants and microorganisms (Fig 3), so fertilisation and particularly liming have been used for nurse grass (M.RG) establishment and peat stabilisation[32] of the highly mobile peat surface[12]. In areas where the bare peat has eroded all the way down to bedrock in gully systems, natural regeneration with acid grass and dwarf shrub communities is taking place (U.Gu). Restoration by spreading heather brash via helicopter over the established nurse grass in the following growth season has been successfully applied on Holme Moss and is expected to work by both stabilising the peat surface and providing plant seed to establish new plants[4, 16]. In fact it is most likely that this practice also delivers a microbial inoculum which may be very beneficial to the process and could be enhanced, for instance by careful selection of source material to include some roots and soil.

Bare peat (D.BP) had a distinct microbial community with reduced richness compared to the other zones (Figs 4 and 6). The complete loss of vegetation and associated primary production from our D.BP zone has not only halted the main photosynthetic carbon input to the soil, but also permitted extensive physical erosion of the surface layers of up to 2 m to occur[12]. As a result we suggest that the un-vegetated bare peat areas have a dysfunctional surface microbial community due to exposure of communities adapted to the underlying saturated and anoxic catotelm horizon, and also exposure of labile carbon which was previously preserved through anoxic and low temperature conditions in this saturated peat horizon[63]. This suggestion could be tested by more targeted studies focusing on specific depths in bare peat and stable vegetated areas. Clearly demonstrating loss of function in bare peat would be useful because modern political and economic developments are increasingly demanding that ecosystems be valued and managed according to the services they provide[64]. We expect that stabilisation of bare peat by any means, including gully blocking and re-vegetation[4], will cut off this supply of buried carbon to the surface, providing an opportunity for establishment of more natural surface microbial populations sustained by input from primary production. Erosion of the bare peat surface has also removed the legacy of anthropogenic heavy metals pollution, leaving a relatively uncontaminated substratum but with ongoing atmospheric N deposition and exposure of buried C[10]. This scenario predicts P limitation in bare peat because there is an ongoing supply of carbon and nitrogen. P limitation is evidenced in our data (Fig 2) and is likely to affect the microbial community structure as recently identified in North-American peatlands[65, 66]. Low fungal richness also identified in 25-year-old heather (M.25) (Fig 4) may reflect the age-related ‘mature’ to early ‘degenerate’ phase of the heather monoculture when productivity declines[67] resulting from protection from fire and stem harvesting or mowing. The loss of active rhizodeposition and increased root senescence leaving recalcitrant lignin-rich litter could be the explanation for reduced fungal richness.

The managed zones of lowland grass (M.RG) and young heather (M.YH) established for 2 and 1 years, respectively, on bare peat (D.BP) all exhibited higher microbial numbers and OTU richness compared to bare peat (Fig 3 and Fig 4). This suggests that natural and early (1–2yr) managed regeneration of bare peat has had a beneficial effect in terms of microbial potential for delivery of ecosystem services below-ground, which may be regarded as a success indicator for restoration. Increased microbial numbers suggest re-establishment of photosynthetic carbon input via root growth and turnover and increased rhizodeposition, all contributing to peat stabilisation and reduced physical erosion[68], whilst increased microbial richness may be driven by the competition and synergies which are expected in the rhizosphere and mycorrhizosphere actively developed in the these early re-vegetated zones[69, 70]. Despite these positive indications it is clear from Figs 5 and 6 that recent restoration effort on former D.BP is associated with a detectable shift in both bacterial and fungal communities (M.RG and M.YH) which are quite different to those in the unmanaged zones and the older restoration (M.25).

Alpha diversity measures give an indication of the number of taxa (richness) or diversity within a population without comparing the presence of particular individuals between populations. Typical measures such as Shannon’s diversity index and the Chao1 richness estimate can be very sensitive to sampling effort, which in our study was lower per sample than has been recommended (5,000 sequences) for microbial alpha diversity comparisons[71]. High levels of variation in bacterial alpha diversity (Chao1, Shannon and Simpson) on a per-sample basis (S3 Fig) are similar to recent findings in other low pH bogs from a UK soil bacterial bio-geography assessment[72]. In their study, bacterial alpha-, beta- and gamma-diversity was determined based on relatively low-resolution 16S TRFLP profiling in comparisons of major UK vegetation zones that included acidic bogs, arable land and alkaline calcareous pastures and dune soils. Soil pH was found to be a major driver of bacterial community structure and the authors recommended more examination of peat bogs at low pH, as carried out in the present study.

Of all the zones studied the elevated original vegetation (U.OV) area exhibited the highest levels of heavy metals pollution (Fig 2) because it has been stable for the longest time, and by this same measure the degraded bare peat (D.BP) may arguably be regarded as the most pristine zone because the polluted surface layers have been eroded away[73].

A previous investigation into the bacterial communities in peat of the southern Pennines concluded that heavy metals are likely to be an important factor influencing bacterial community structure[25]. We found that the original vegetation (U.OV) microbial community remains distinct from the other zones for both bacteria and fungi, and this may be related to the fact that this stable area has retained deposited heavy metals. Under present reduced heavy metal deposition[10] it therefore seems unlikely that any restoration from relatively pristine bare peat will reach the same U.OV climax bacterial and fungal community composition because of the reduced heavy metals loadings. This raises questions as to the potential functional effect the legacy of heavy metals pollution might be having on the belowground microbial communities in long-term stable peatland areas that appear above-ground to be in good condition. It is quite feasible that heavy metals pollution may have reduced belowground microbial capacity for supporting the above-ground vegetation in such a way as to have contributed to the degradation of the blanket bog.

Natural re-vegetation in the gully (U.Gu) is completely different to the managed restorations of bare peat. Here, no more peat erosion is possible beyond the exposed gritstone bedrock so there is no direct carbon input from buried peat stocks, but there is abundant water carrying dissolved and particulate carbon into these gullies[12]. High levels of P and nitrate support growth of acid grasses but also dwarf shrubs and sedges that are linked with large and rich microbial communities (Fig 3 and Fig 4). Of all the zones the gully is most similar to the majority of previous studies on peatland microbial communities, being saturated much of the time and even supporting some *Sphagnum* mosses[20, 21]. The gully environment greatly differs from ombrotrophic bog in that nutrients including P can arise from bedrock weathering much as in minerotrophic fens that are significantly more fertile in relation to N, P, K, and Ca, as recently reviewed[74]. Additional input could be occurring from restoration derived fertiliser discharge into the gullies.

## Conclusions

We show that six zones encompassing degraded bare peat and vegetation mosaics in an upland peatland support distinct microbial communities, which can be linked to natural processes and human intervention in the management of peatlands. Microbial community evidence suggests that degraded bare peat may be functionally impaired, and that re-vegetation by natural or managed means could restore functional potential in the soil microbiome. None of the re-vegetated zones established a microbial community resembling the original dwarf shrub vegetation even after 25 years, and this may in part be due to a legacy of pollution that is stabilised in the original vegetation zones. We suggest that the outlook for ecosystem function in natural and managed re-vegetated bare peat at Holme Moss and similar industrially impacted sites is good because the exposed subsurface peat is essentially pristine. This is in contrast to the long-term stabilised original vegetation zones supporting dwarf shrub communities that will continue to be impacted by historical pollution for the foreseeable future, thus re-vegetated bare peat areas may well achieve greater biodiversity and ecosystem functionality.

## Supporting Information

S1 Protocol Data and source code sufficient to reproduce the analyses presented in this paper. UPARSE clusters, dereplicated DNA sequences, full taxonomic assignments, OTU abundance tables and sample data are provided. Outputs from the analyses are also provided, including statistical tables and the number of sequences for each sample.
(ZIP)

S1 Table Sample metadata and results. Sample locations, physico-chemical data, and cultivable microbes from bare peat and the five vegetated zones (see Table 1) at Holme Moss.
(DOCX)

S2 Table Mean relative abundance of phyla in each zone. Relative abundances are expressed as a percentage within each kingdom (i.e. columns add up to 200%).
(DOCX)

S3 Table Pairwise comparisons of phylum abundance in bare peat (D.BP) and original vegetation (U.OV) zones. (DOCX)

S4 Table Mann-Whitney-Wilcoxon test results for various comparisons of chemistry and cultivable microbe data. (DOCX)

S1 Fig Unconstrained correspondence analyses of microbial communities in bare and vegetated peat. Analysis is based on relative abundance of 354 bacterial OTUS and 273 fungal OTUs across six zone classifications (Table 1). Markers indicate individual samples (three per zone type). Scree plots are provided in S2 Fig.
(TIF)

S2 Fig Scree plots for constrained and unconstrained ordination of microbial communities (Fig 6 and S1 Fig) (TIF)

S3 Fig Diversity metrics for each zone, per-sample and per-zone. Based on 3 samples per zone analysed separately (mean 1742 sequences), and together (mean 5460 sequences).
(TIF)

S4 Fig Pre-restoration state of degraded ombrotrophic peatland at Holme Moss. Note bare unconsolidated peat areas, cotton grass and dwarf shrub dominated gully vegetation and elevated remnants of the peat dome supporting original dwarf shrub vegetation. Photograph taken July 2006.
(JPG)

S5 Fig Pre-restoration state of degraded ombrotrophic peatland at Holme Moss. Note bare unconsolidated peat gully walls and exposed gritstone bedrock margins in a gully supporting naturally regenerated cotton grass/grass/dwarf shrub dominated vegetation and elevated remnants of the peat dome supporting original dwarf shrub vegetation in the background. Photograph taken July 2006.
(JPG)
