# Supplementary material for: Bacterial and Fungal Communities in a Degraded Ombrotrophic Peatland Undergoing Natural and Managed Re-Vegetation
Source: PLoS One. 2015 May 13;10(5):e0124726. doi: 10.1371/journal.pone.0124726 (PMC4430338; doi:10.1371/journal.pone.0124726)
Supplement: S1 Protocol — UPARSE clusters, dereplicated DNA sequences, full taxonomic assignments, OTU abundance tables and sample data are provided. Outputs from the analyses are also provided, including statistical tables and the number of sequences for each sample. (ZIP) [file pone.0124726.s001.zip › S1_Protocol/peat-microbiome.html]

- Bacterial and fungal communities in a degraded ombotrophic peatland undergoing natural and managed re-vegetation
  - Key to zone codes
  - Source code
  - Source data
  - Figures and Tables
  - Calculations for manuscript text
  - R package versions
  - Citations

# Bacterial and fungal communities in a degraded ombotrophic peatland undergoing natural and managed re-vegetation

## Key to zone codes

- U.OV Unmanaged - original vegetation
- U.Gu Unamanaged - gully
- M.YH Managed - young heather
- M.RG Managed - restored grass
- M.25 Managed - 25 year old heather
- D.BP Degraded - bare peat

## Source code

Analyses were performed in R. Commented source code is provided in file peat-microbiome.Rmd.

## Source data

There were 94627 bacterial and 84552 fungal raw reads off the sequencer. UPARSE clustered OTUs and BLAST assigned taxonomy were imported and analysed using the phyloseq package for R. The source data for bacteria and fungi can be found in folders data/hb\_uparse and data/hf\_uparse respectively. Those folders also contain a record of commands used to prepare the raw sequences to this stage.

```
print(expt.bacteria)
```

```
## phyloseq-class experiment-level object
## otu_table()   OTU Table:         [ 441 taxa and 18 samples ]
## sample_data() Sample Data:       [ 18 samples by 27 sample variables ]
## tax_table()   Taxonomy Table:    [ 441 taxa by 7 taxonomic ranks ]
```

```
print(expt.fungi)
```

```
## phyloseq-class experiment-level object
## otu_table()   OTU Table:         [ 300 taxa and 18 samples ]
## sample_data() Sample Data:       [ 18 samples by 27 sample variables ]
## tax_table()   Taxonomy Table:    [ 300 taxa by 7 taxonomic ranks ]
```

Number of quality controlled reads per sample:

```
colSums(otu_table(expt.bacteria))
```

```
##   W1   W2   W3   W4   W5   W6   M1   M2   M3   M4   M5   M6   E1   E2   E3 
## 1469  373 2233 3150 1695 2072 2046 2398 3968 1714 3435  992 1489 1954 1169 
##   E4   E5   E6 
##  398 1117 1088
```

```
colSums(otu_table(expt.fungi))
```

```
##   W1   W2   W3   W4   W5   W6   M1   M2   M3   M4   M5   M6   E1   E2   E3 
## 2825  705 2114 3464  724 1333 3319 1688 2096  328 2072 1369 1136  708 1799 
##   E4   E5   E6 
## 1859  932 1491
```

Number of quality controlled reads per zone:

```
rowSums(otu_table(expt.bacteria.zone))
```

```
## D.BP M.RG M.YH M.25 U.Gu U.OV 
## 3940 4641 6599 5004 7702 4874
```

```
rowSums(otu_table(expt.fungi.zone))
```

```
## D.BP M.RG M.YH M.25 U.Gu U.OV 
## 4192 2801 6069 7280 6468 3152
```

## Figures and Tables

pdf 2

pdf 2 pdf 2 pdf 2

```
## [1] TRUE
```

  
> **Figure 2. [chem].** Plant nutrients, soil properties, and heavy metals.

---

  
> **Figure 3. [cfu].** Cultivable numbers of bacteria and fungi detected in each zone.

---

  
> **Figure 4. [rankf].** Rank abundance of bacterial and fungal OTUs in each zone.

---

  
> **Figure 5. [phyla].** Relative abundance of bacterial and fungal phyla (within each kingdom) in the six zones.

---

  
> **Figure 6. [ord].** Correspondence analysis of bacterial and fungal communities, constrained by vegetation zone.

---

  
> |  | Kingdom | Phylum | D.BP | M.25 | M.RG | M.YH | U.Gu | U.OV |
> | --- | --- | --- | --- | --- | --- | --- | --- | --- |
> | B2 | Bacteria | Acidobacteria | 33.92 | 38.57 | 19.80 | 19.82 | 43.49 | 28.72 |
> | B1 | Bacteria | Proteobacteria | 49.57 | 49.93 | 55.49 | 48.95 | 44.88 | 51.32 |
> | B37 | Bacteria | Bacteroidetes | 0.79 | 3.32 | 2.89 | 2.22 | 2.72 | 4.49 |
> | B443 | Bacteria | Firmicutes | 0.03 | 0.09 | 0.00 | 0.00 | 0.00 | 0.06 |
> | B310 | Bacteria | OD1 | 0.00 | 0.05 | 0.05 | 0.00 | 0.00 | 0.00 |
> | B10 | Bacteria | Actinobacteria | 9.47 | 5.55 | 11.63 | 12.04 | 4.70 | 10.82 |
> | B92 | Bacteria | TM7 | 0.00 | 0.11 | 0.10 | 0.08 | 0.07 | 0.29 |
> | B166 | Bacteria | Spirochaetes | 0.00 | 0.29 | 0.02 | 0.03 | 0.09 | 0.00 |
> | B7 | Bacteria | AD3 | 2.91 | 0.22 | 5.62 | 11.43 | 1.53 | 2.29 |
> | B50 | Bacteria | Verrucomicrobia | 1.17 | 0.73 | 1.82 | 3.26 | 0.96 | 0.43 |
>
> Table was truncated to 10 rows for display. Click link to see whole table
>
> **Table S3. [all\_phyla].** Mean relative abundance of all phyla in each zone. Relative abundances are expressed as a percentage within each kingdom (i.e. columns add up to 200 %)

---

  
> |  | Phylum | D.BP | U.OV | D.BP.expected | U.OV.expected | Xsq\_p.value | Xsq\_p.value.corrected | D.BP\_pc | U.OV\_pc | diff |
> | --- | --- | --- | --- | --- | --- | --- | --- | --- | --- | --- |
> | F20 | Zygomycota | 58.00 | 557.00 | 351.05 | 263.95 | 0.00 | 0.00 | 1.38 | 17.67 | -16.29 |
> | F1 | Ascomycota | 2121.00 | 1882.00 | 2284.94 | 1718.06 | 0.00 | 0.00 | 50.60 | 59.71 | -9.11 |
> | B37 | Bacteroidetes | 27.00 | 204.00 | 103.26 | 127.74 | 0.00 | 0.00 | 0.69 | 4.19 | -3.50 |
> | B10 | Actinobacteria | 380.00 | 546.00 | 413.94 | 512.06 | 0.02 | 0.04 | 9.64 | 11.20 | -1.56 |
> | B1 | Proteobacteria | 1980.00 | 2483.00 | 1995.03 | 2467.97 | 0.65 | 0.65 | 50.25 | 50.94 | -0.69 |
> | B165 | WPS-2 | 29.00 | 27.00 | 25.03 | 30.97 | 0.29 | 0.33 | 0.74 | 0.55 | 0.18 |
> | B81 | TM6 | 29.00 | 7.00 | 16.09 | 19.91 | 0.00 | 0.00 | 0.74 | 0.14 | 0.59 |
> | B7 | AD3 | 115.00 | 112.00 | 101.47 | 125.53 | 0.07 | 0.09 | 2.92 | 2.30 | 0.62 |
> | B39 | Verrucomicrobia | 44.00 | 19.00 | 28.16 | 34.84 | 0.00 | 0.00 | 1.12 | 0.39 | 0.73 |
> | F64 | Glomeromycota | 41.00 | 5.00 | 26.26 | 19.74 | 0.00 | 0.00 | 0.98 | 0.16 | 0.82 |
>
> Table was truncated to 10 rows for display. Click link to see whole table
>
> **Table S4. [phyla-xsq].** Pairwise comparisons of phylum abundance in D.BP and U.OV. Note that p-values of 0 in the linked file actually mean <2.2e-16.

---

  
> **Figure S5. [ord\_unconstrained].** Unconstrained correspondence analyses.

---

  
> **Figure S6. [scree].** Scree plots for constrained and unconstrained ordinations

---

  
> |  | variable | factor | W | P |
> | --- | --- | --- | --- | --- |
> | 1 | pH | bare peat and early stage restoration/other | 31 | 0.42582 |
> | 2 | Moisture | bare peat and early stage restoration/other | 81 | 0.00004 |
> | 3 | OM | bare peat and early stage restoration/other | 81 | 0.00004 |
> | 4 | Ammonium | bare peat and early stage restoration/other | 18 | 0.05031 |
> | 5 | Nitrate | bare peat and early stage restoration/other | 30 | 0.38651 |
> | 6 | P | bare peat and early stage restoration/other | 8 | 0.00276 |
> | 7 | K | bare peat and early stage restoration/other | 16 | 0.03147 |
> | 8 | Pb | bare peat and early stage restoration/other | 1 | 0.00008 |
> | 9 | Cd | bare peat and early stage restoration/other | 6 | 0.00123 |
> | 10 | Cu | bare peat and early stage restoration/other | 2 | 0.00016 |
>
> Table was truncated to 10 rows for display. Click link to see whole table
>
> **Table S7. [stats].** Mann-Whitney-Wilcoxon test results for various comparisons of chemistry and cultivable microbe data.

---

  
> **Figure S8. [diversity].** Diversity metrics for each zone, per-sample and per-zone

---

## Calculations for manuscript text

```
## $`n archaeal observations`
## [1] 2
## 
## $`n bacterial observations`
## [1] 32760
## 
## $`n fungal observations`
## [1] 29962
## 
## $`n archael OTUs`
## [1] 1
## 
## $`n bacterial OTUs`
## [1] 441
## 
## $`n fungal OTUs`
## [1] 300
## 
## $`n bacterial phyla`
## [1] 21
## 
## $`n fungal phyla`
## [1] 5
## 
## $`mean seqs per sample fungi`
## [1] 1665
## 
## $`mean seqs per sample bacteria`
## [1] 1820
## 
## $`mean seqs per sample per kingdom`
## [1] 1742
## 
## $`mean seqs per zone fungi`
## [1] 4995
## 
## $`mean seqs per zone bacteria`
## [1] 5460
## 
## $`mean seqs per zone per kingdom`
## [1] 5226
## 
## $`mean pH all samples`
## [1] 3.878
```

## R package versions

```
print(sessionInfo(), locale = FALSE)
```

```
## R version 3.1.1 (2014-07-10)
## Platform: x86_64-w64-mingw32/x64 (64-bit)
## 
## attached base packages:
## [1] grid      stats     graphics  grDevices utils     datasets  methods  
## [8] base     
## 
## other attached packages:
## [1] car_2.0-21      vegan_2.0-10    lattice_0.20-29 permute_0.8-3  
## [5] xtable_1.7-3    reshape2_1.4    ggplot2_1.0.0   phyloseq_1.9.11
## 
## loaded via a namespace (and not attached):
##  [1] ade4_1.6-2              annotate_1.40.1        
##  [3] AnnotationDbi_1.24.0    ape_3.1-4              
##  [5] Biobase_2.22.0          BiocGenerics_0.8.0     
##  [7] biom_0.3.12             Biostrings_2.30.1      
##  [9] cluster_1.15.2          codetools_0.2-8        
## [11] colorspace_1.2-4        data.table_1.9.2       
## [13] DBI_0.2-7               DESeq2_1.4.5           
## [15] digest_0.6.4            evaluate_0.5.5         
## [17] foreach_1.4.2           formatR_0.10           
## [19] genefilter_1.44.0       geneplotter_1.40.0     
## [21] GenomicRanges_1.14.4    gtable_0.1.2           
## [23] htmltools_0.2.4         igraph_0.7.1           
## [25] IRanges_1.20.7          iterators_1.0.7        
## [27] knitr_1.6               labeling_0.2           
## [29] locfit_1.5-9.1          MASS_7.3-33            
## [31] Matrix_1.1-4            multtest_2.18.0        
## [33] munsell_0.4.2           nlme_3.1-117           
## [35] nnet_7.3-8              parallel_3.1.1         
## [37] plyr_1.8.1              proto_0.3-10           
## [39] RColorBrewer_1.0-5      Rcpp_0.11.2            
## [41] RcppArmadillo_0.4.320.0 RJSONIO_1.2-0.2        
## [43] rmarkdown_0.2.50        RSQLite_0.11.4         
## [45] scales_0.2.4            splines_3.1.1          
## [47] stats4_3.1.1            stringr_0.6.2          
## [49] survival_2.37-7         tools_3.1.1            
## [51] XML_3.98-1.1            XVector_0.2.0          
## [53] yaml_2.1.13
```

## Citations

```
citation("vegan")
```

```
## 
## To cite package 'vegan' in publications use:
## 
##   Jari Oksanen, F. Guillaume Blanchet, Roeland Kindt, Pierre
##   Legendre, Peter R. Minchin, R. B. O'Hara, Gavin L. Simpson,
##   Peter Solymos, M. Henry H. Stevens and Helene Wagner (2013).
##   vegan: Community Ecology Package. R package version 2.0-10.
##   http://CRAN.R-project.org/package=vegan
## 
## A BibTeX entry for LaTeX users is
## 
##   @Manual{,
##     title = {vegan: Community Ecology Package},
##     author = {Jari Oksanen and F. Guillaume Blanchet and Roeland Kindt and Pierre Legendre and Peter R. Minchin and R. B. O'Hara and Gavin L. Simpson and Peter Solymos and M. Henry H. Stevens and Helene Wagner},
##     year = {2013},
##     note = {R package version 2.0-10},
##     url = {http://CRAN.R-project.org/package=vegan},
##   }
## 
## ATTENTION: This citation information has been auto-generated from
## the package DESCRIPTION file and may need manual editing, see
## 'help("citation")'.
```

```
citation("phyloseq")
```

```
## 
## To cite phyloseq in publications, or otherwise credit, please use:
## 
##   phyloseq: An R package for reproducible interactive analysis and
##   graphics of microbiome census data. Paul J. McMurdie and Susan
##   Holmes (2013) PLoS ONE 8(4):e61217.
## 
## A BibTeX entry for LaTeX users is
## 
##   @Article{,
##     author = {Paul J. McMurdie and Susan Holmes},
##     journal = {PLoS ONE},
##     pages = {e61217},
##     title = {phyloseq: An R package for reproducible interactive analysis and graphics of microbiome census data},
##     volume = {8},
##     number = {4},
##     year = {2013},
##     url = {http://dx.plos.org/10.1371/journal.pone.0061217},
##   }
```
