# Supplementary material for: Bacterial and Fungal Communities in a Degraded Ombrotrophic Peatland Undergoing Natural and Managed Re-Vegetation
Source: PLoS One. 2015 May 13;10(5):e0124726. doi: 10.1371/journal.pone.0124726 (PMC4430338; doi:10.1371/journal.pone.0124726)
Supplement: S1 Protocol — UPARSE clusters, dereplicated DNA sequences, full taxonomic assignments, OTU abundance tables and sample data are provided. Outputs from the analyses are also provided, including statistical tables and the number of sequences for each sample. (ZIP) [file pone.0124726.s001.zip › S1_Protocol/README.html]

Record of analysis. R scripts and source data for article submitted to PLoS ONE:

### DR Elliott, SJ Caporn, F Nwaishi, RH Nilsson, R Sen

#### Bacterial and fungal communities in a degraded ombotrophic peatland undergoing natural and managed re-vegetation

The UK hosts 15-19% of global upland ombotrophic (rain fed) peatlands that are estimated to store 3.2 billion tonnes of carbon and represent a critical upland habitat with regard to biodiversity and ecosystem services provision. Net production is dependent on an imbalance between growth of peat-forming Sphagnum mosses and microbial decomposition by microorganisms that are limited by cold, acidic, and anaerobic conditions. In the Southern Pennines, land-use change, drainage, and over 200 years of anthropogenic N and heavy metal deposition have contributed to severe peatland degradation manifested as a loss of vegetation leaving bare peat susceptible to erosion and deep gullying. A restoration programme designed to regain peat hydrology, stability and functionality has involved re-vegetation through nurse grass, dwarf shrub and Sphagnum re-introduction.

Our aim was to characterise bacterial and fungal communities, via high-throughput rRNA gene sequencing, in the surface acrotelm/mesotelm of degraded bare peat, long-term stable vegetated peat, and natural and managed restorations. Compared to long-term vegetated areas the bare peat microbiome had significantly higher levels of oligotrophic marker phyla (*Acidobacteria*, *Verrucomicrobia*, TM6) and lower *Bacteroidetes* and *Actinobacteria*, together with much higher ligninolytic *Basidiomycota*. Fewer distinct OTUs and significantly fewer cultivable microbes were detected in bare peat compared to other areas. Microbial community structure was linked to restoration activity and correlated with soil edaphic variables (e.g. moisture and heavy metals). Although rapid community changes were evident following restoration activity, restored bare peat did not approach a similar microbial community structure to non-eroded areas even after 25 years, which may be related to the stabilisation of historic deposited heavy metals pollution in long-term stable areas. These primary findings are discussed in relation to bare peat oligotrophy, re-vegetation recalcitrance, rhizosphere-microbe-soil interactions, C, N and P cycling, trajectory of restoration, and ecosystem service implications for peatland restoration.

- Bacterial data
- Fungal data
- Analysis script
