# Supplementary figures and images for: Bacterial and Fungal Communities in a Degraded Ombrotrophic Peatland Undergoing Natural and Managed Re-Vegetation
Source: PLoS One. 2015 May 13;10(5):e0124726. doi: 10.1371/journal.pone.0124726 (PMC4430338; doi:10.1371/journal.pone.0124726)

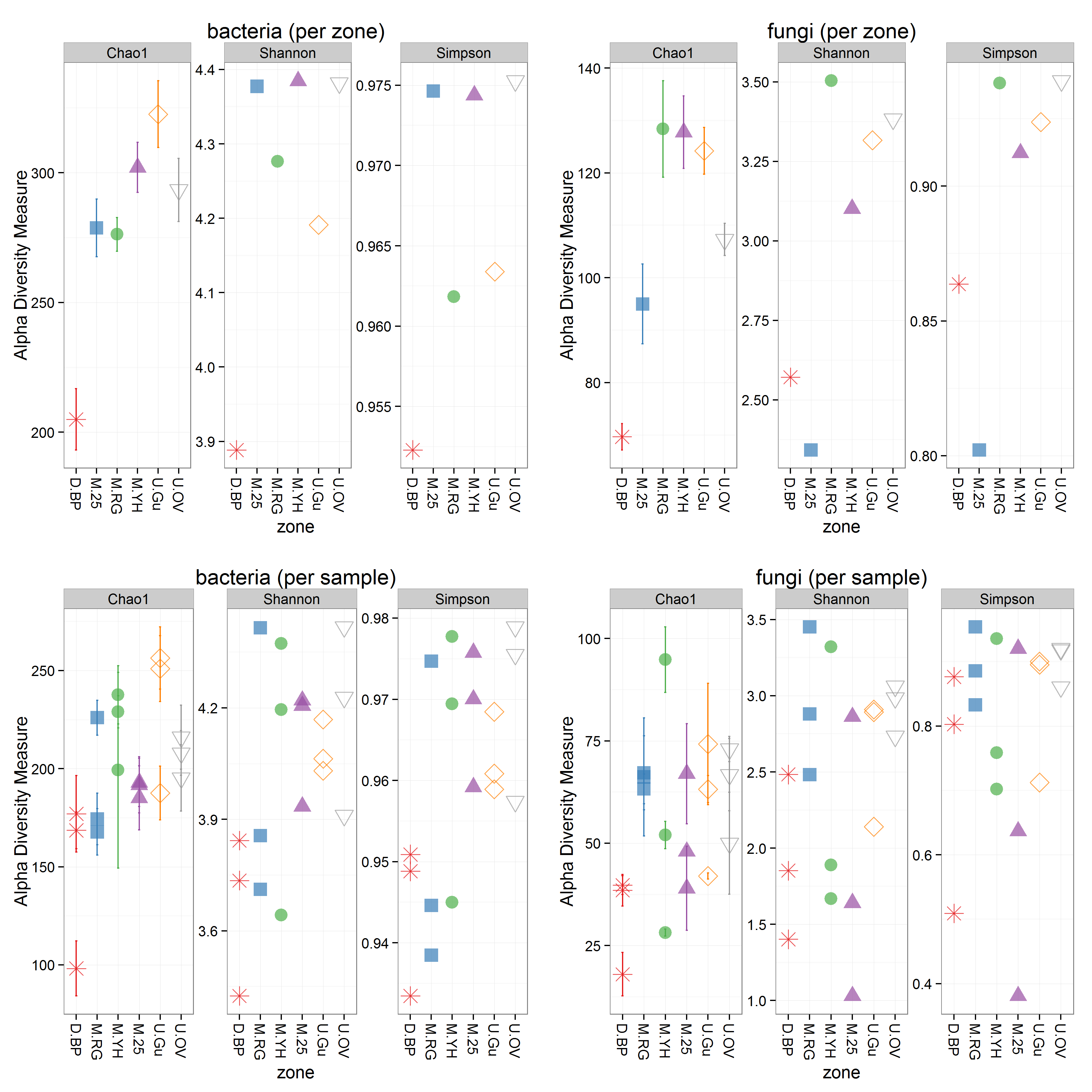

Supplement: S1 Protocol — UPARSE clusters, dereplicated DNA sequences, full taxonomic assignments, OTU abundance tables and sample data are provided. Outputs from the analyses are also provided, including statistical tables and the number of sequences for each sample. (ZIP) [file pone.0124726.s001.zip › S1_Protocol/output/figures/diversity.png]

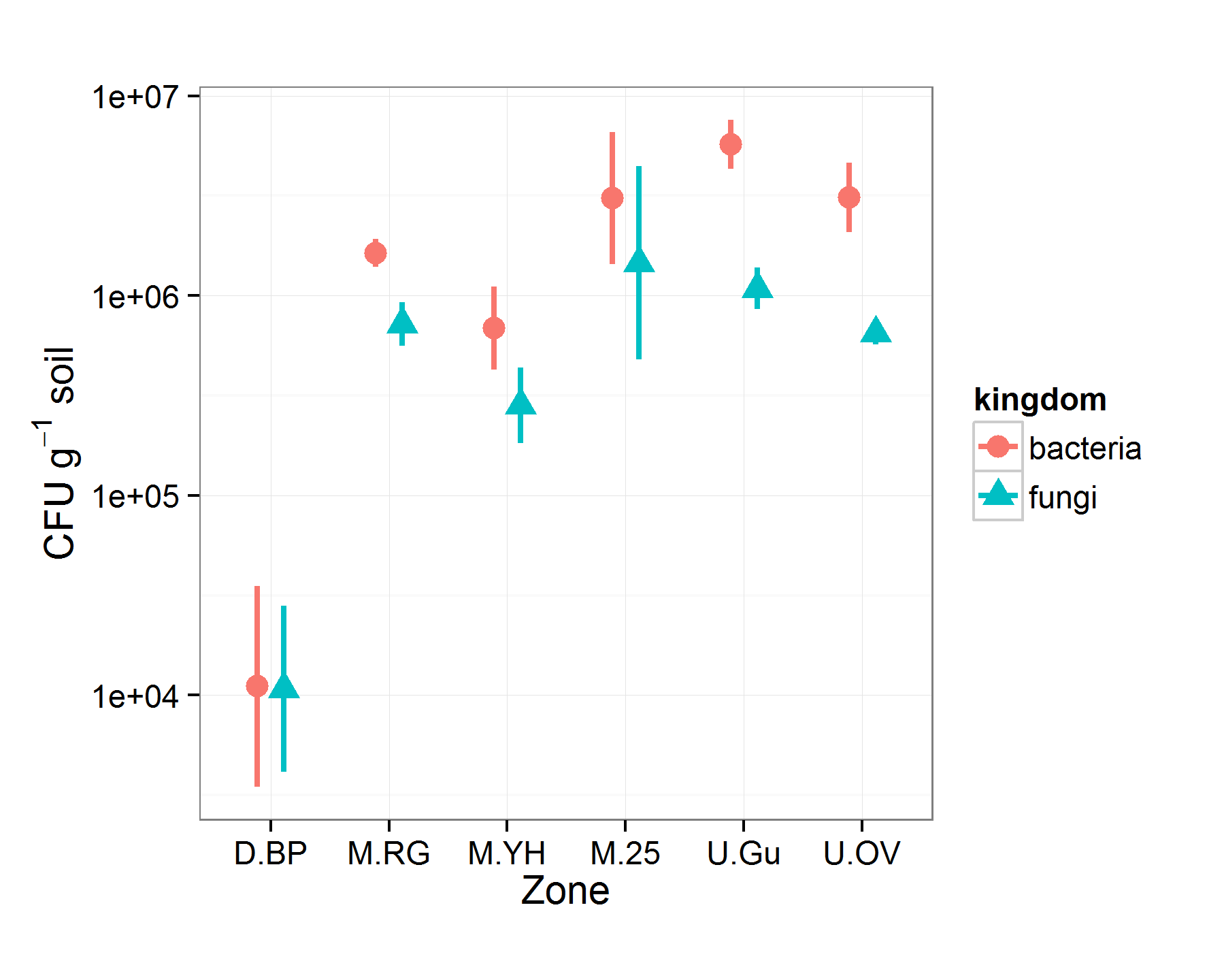

Supplement: S1 Protocol — UPARSE clusters, dereplicated DNA sequences, full taxonomic assignments, OTU abundance tables and sample data are provided. Outputs from the analyses are also provided, including statistical tables and the number of sequences for each sample. (ZIP) [file pone.0124726.s001.zip › S1_Protocol/output/figures/fig[cfu].png]

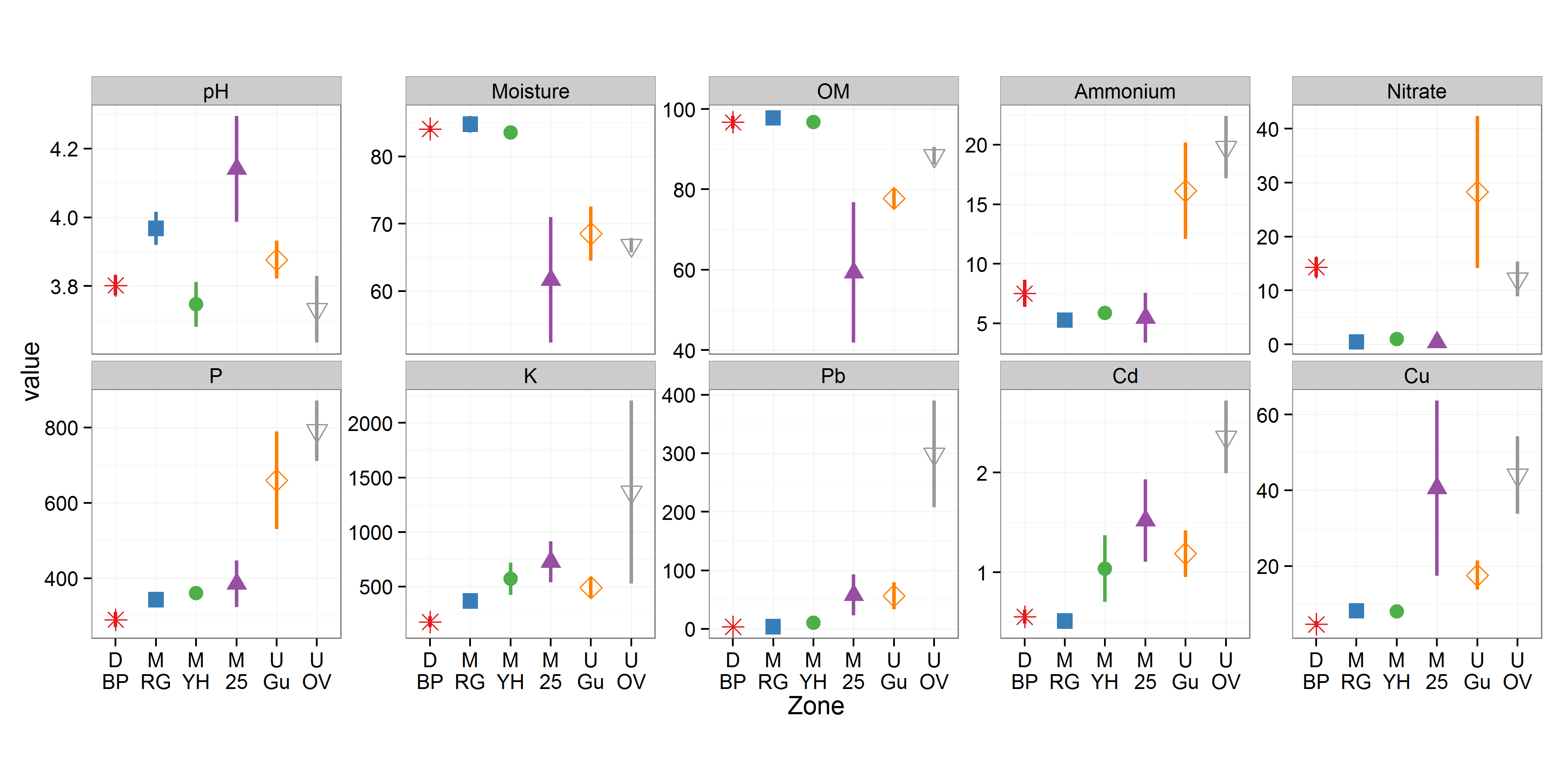

Supplement: S1 Protocol — UPARSE clusters, dereplicated DNA sequences, full taxonomic assignments, OTU abundance tables and sample data are provided. Outputs from the analyses are also provided, including statistical tables and the number of sequences for each sample. (ZIP) [file pone.0124726.s001.zip › S1_Protocol/output/figures/fig[chem].png]

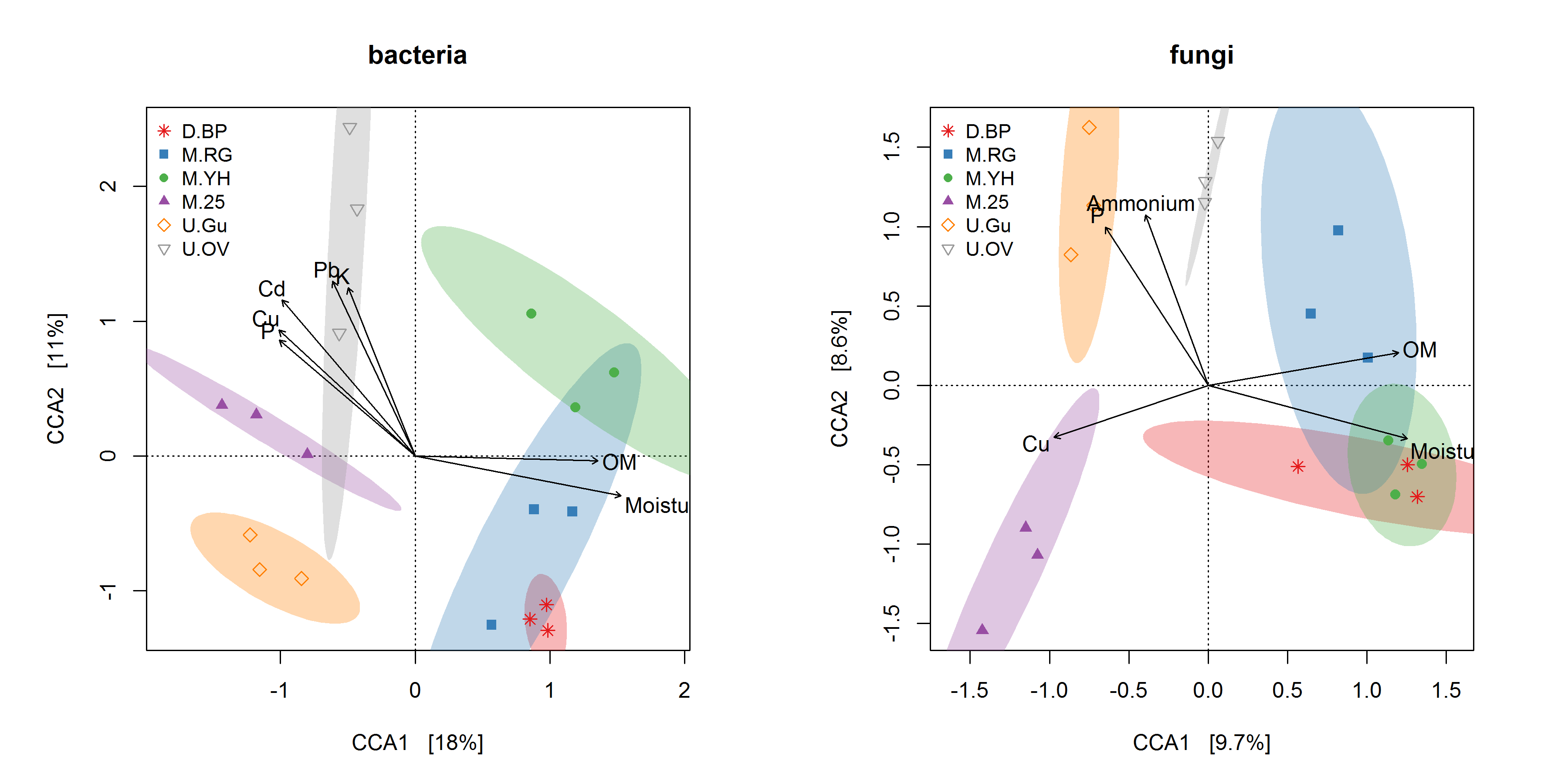

Supplement: S1 Protocol — UPARSE clusters, dereplicated DNA sequences, full taxonomic assignments, OTU abundance tables and sample data are provided. Outputs from the analyses are also provided, including statistical tables and the number of sequences for each sample. (ZIP) [file pone.0124726.s001.zip › S1_Protocol/output/figures/Fig[ordination].png]

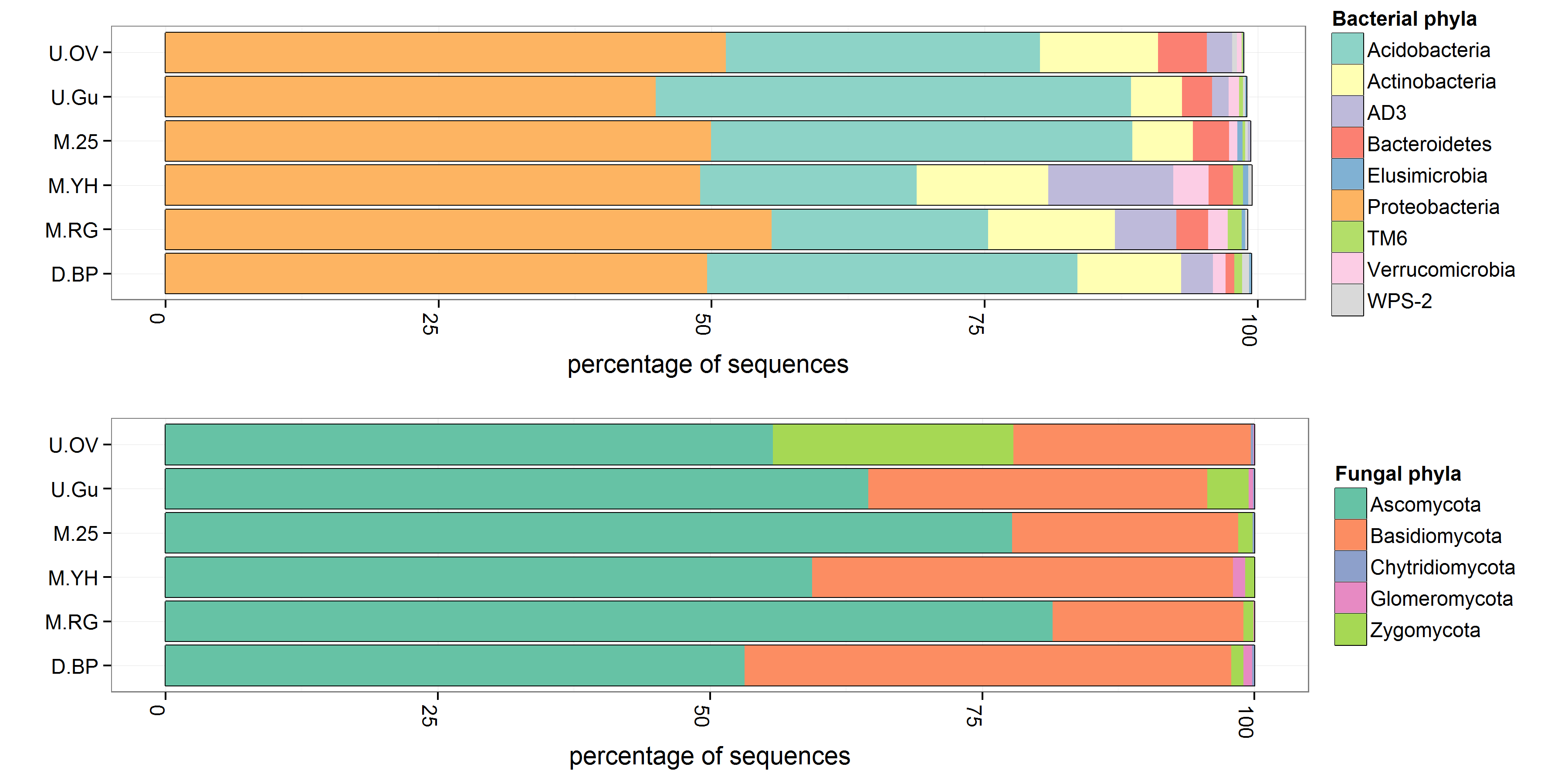

Supplement: S1 Protocol — UPARSE clusters, dereplicated DNA sequences, full taxonomic assignments, OTU abundance tables and sample data are provided. Outputs from the analyses are also provided, including statistical tables and the number of sequences for each sample. (ZIP) [file pone.0124726.s001.zip › S1_Protocol/output/figures/fig[phyla].png]

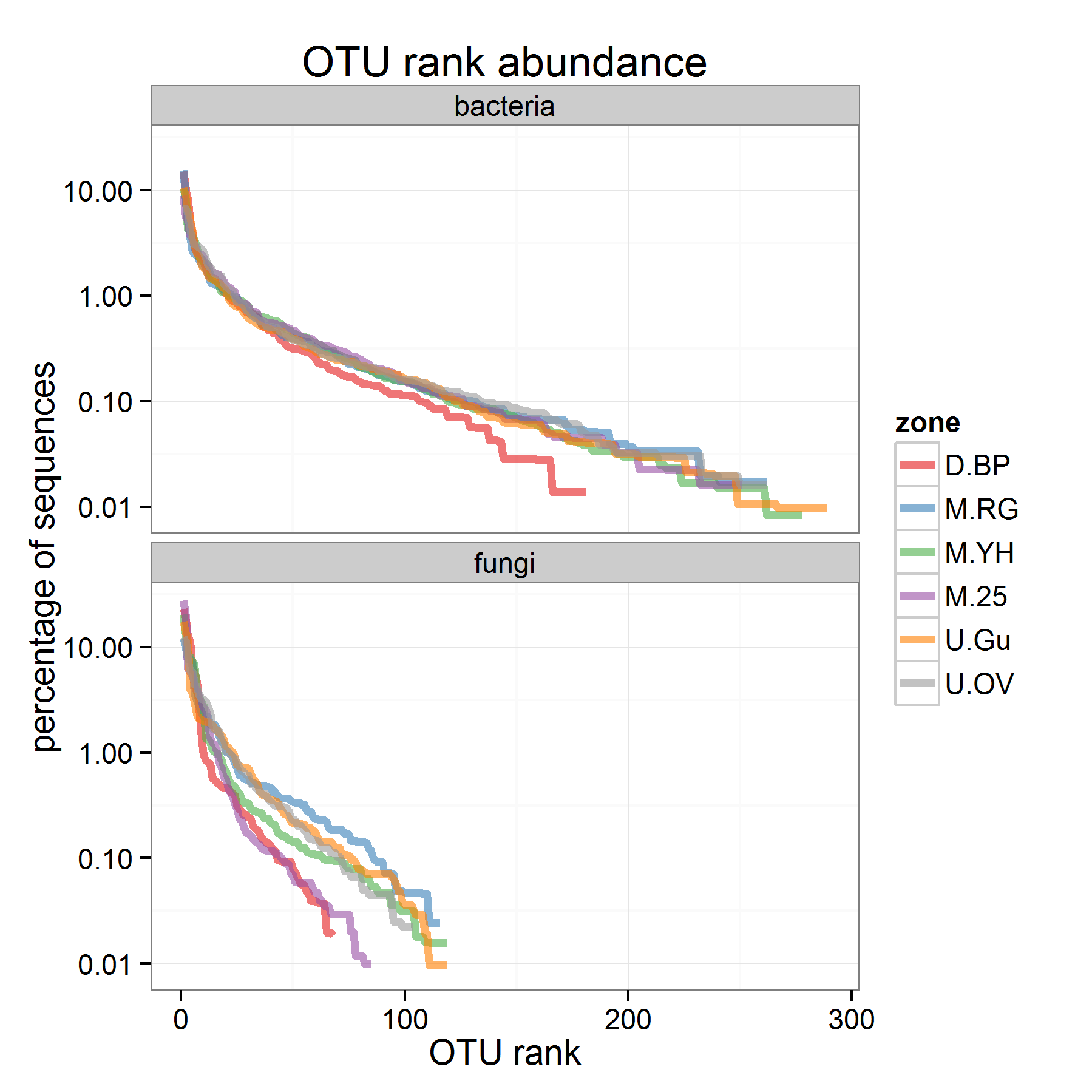

Supplement: S1 Protocol — UPARSE clusters, dereplicated DNA sequences, full taxonomic assignments, OTU abundance tables and sample data are provided. Outputs from the analyses are also provided, including statistical tables and the number of sequences for each sample. (ZIP) [file pone.0124726.s001.zip › S1_Protocol/output/figures/fig[rank].png]

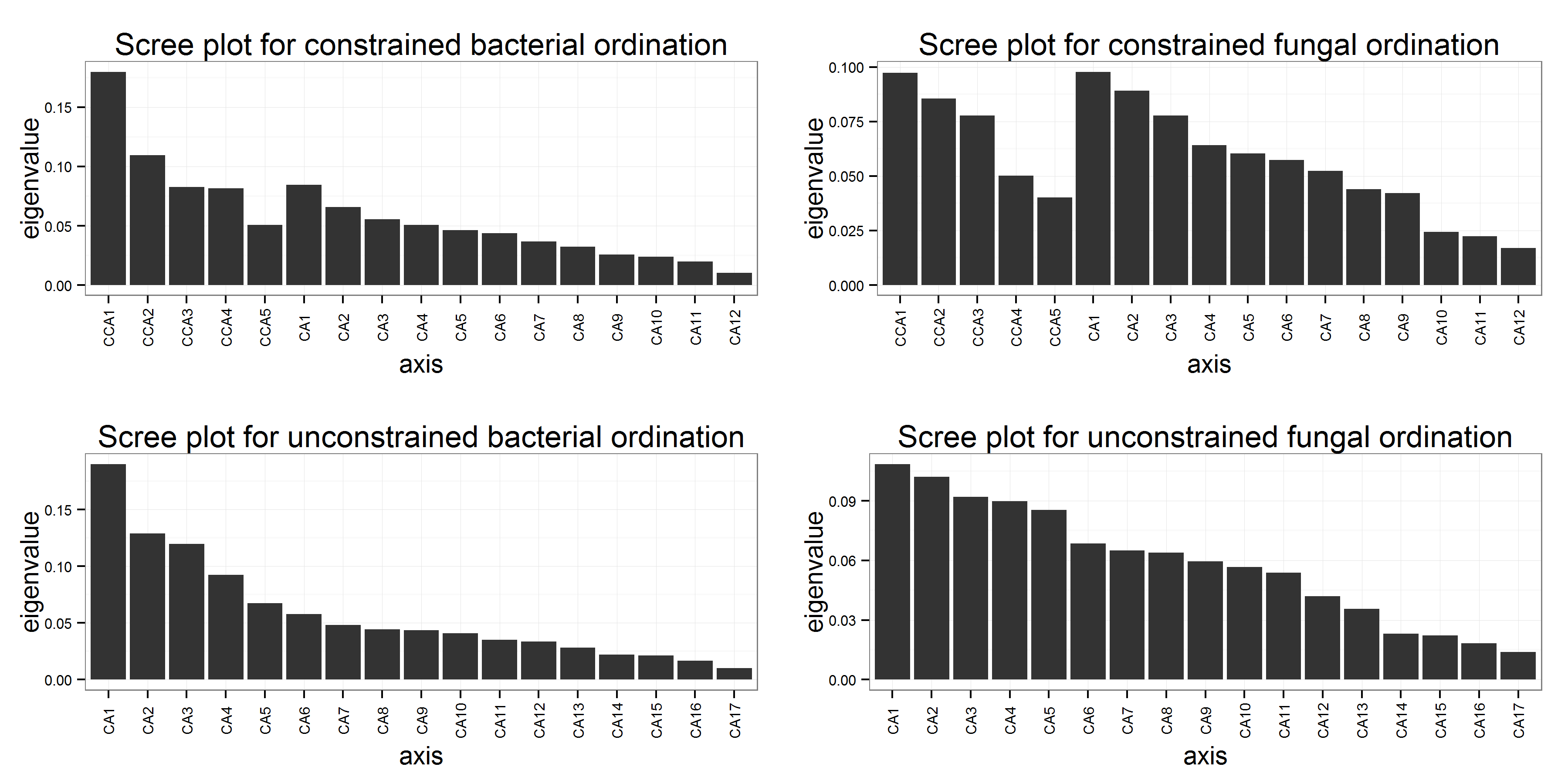

Supplement: S1 Protocol — UPARSE clusters, dereplicated DNA sequences, full taxonomic assignments, OTU abundance tables and sample data are provided. Outputs from the analyses are also provided, including statistical tables and the number of sequences for each sample. (ZIP) [file pone.0124726.s001.zip › S1_Protocol/output/figures/scree.png]

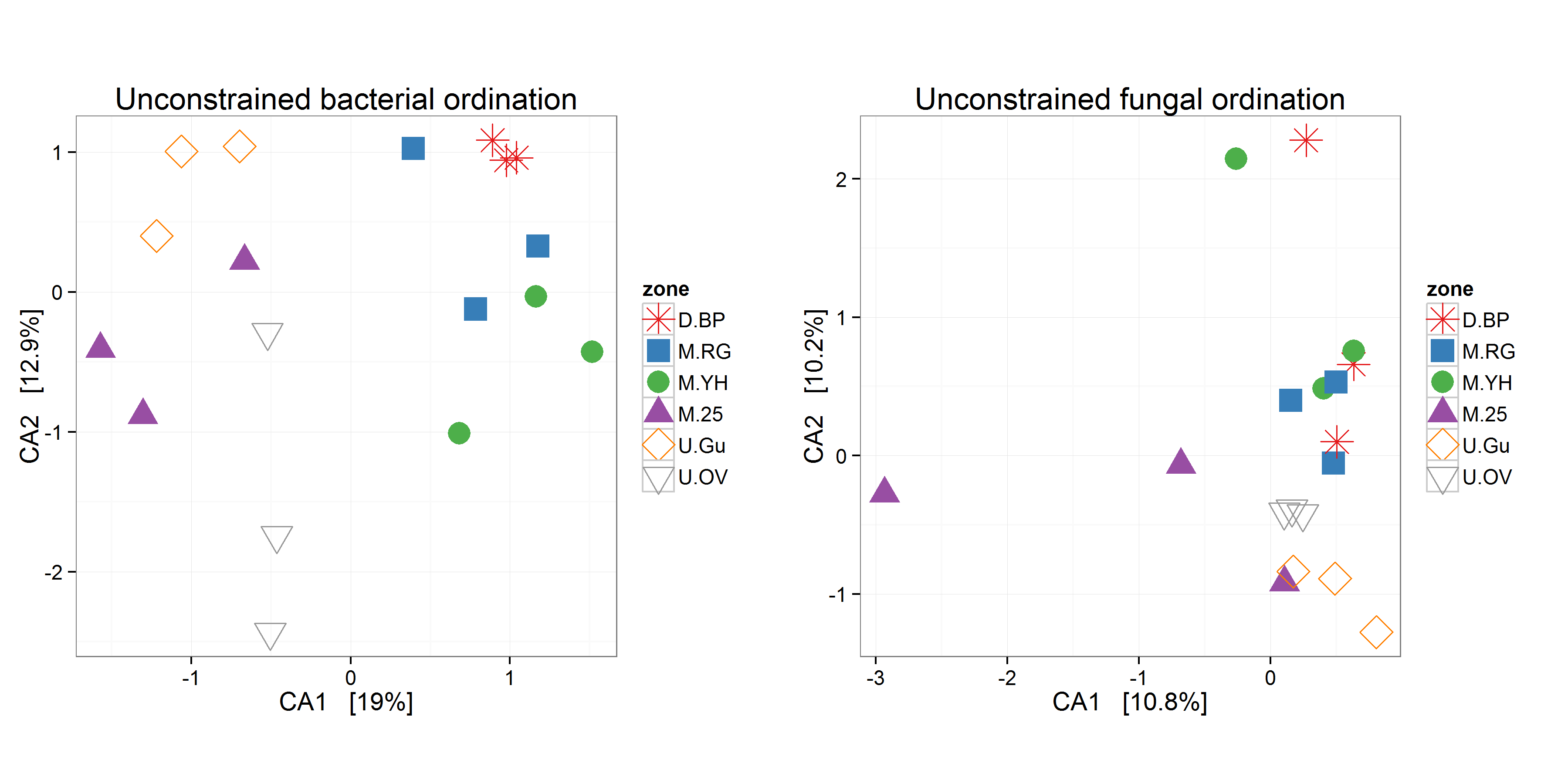

Supplement: S1 Protocol — UPARSE clusters, dereplicated DNA sequences, full taxonomic assignments, OTU abundance tables and sample data are provided. Outputs from the analyses are also provided, including statistical tables and the number of sequences for each sample. (ZIP) [file pone.0124726.s001.zip › S1_Protocol/output/figures/unconstrained_ordinations.png]

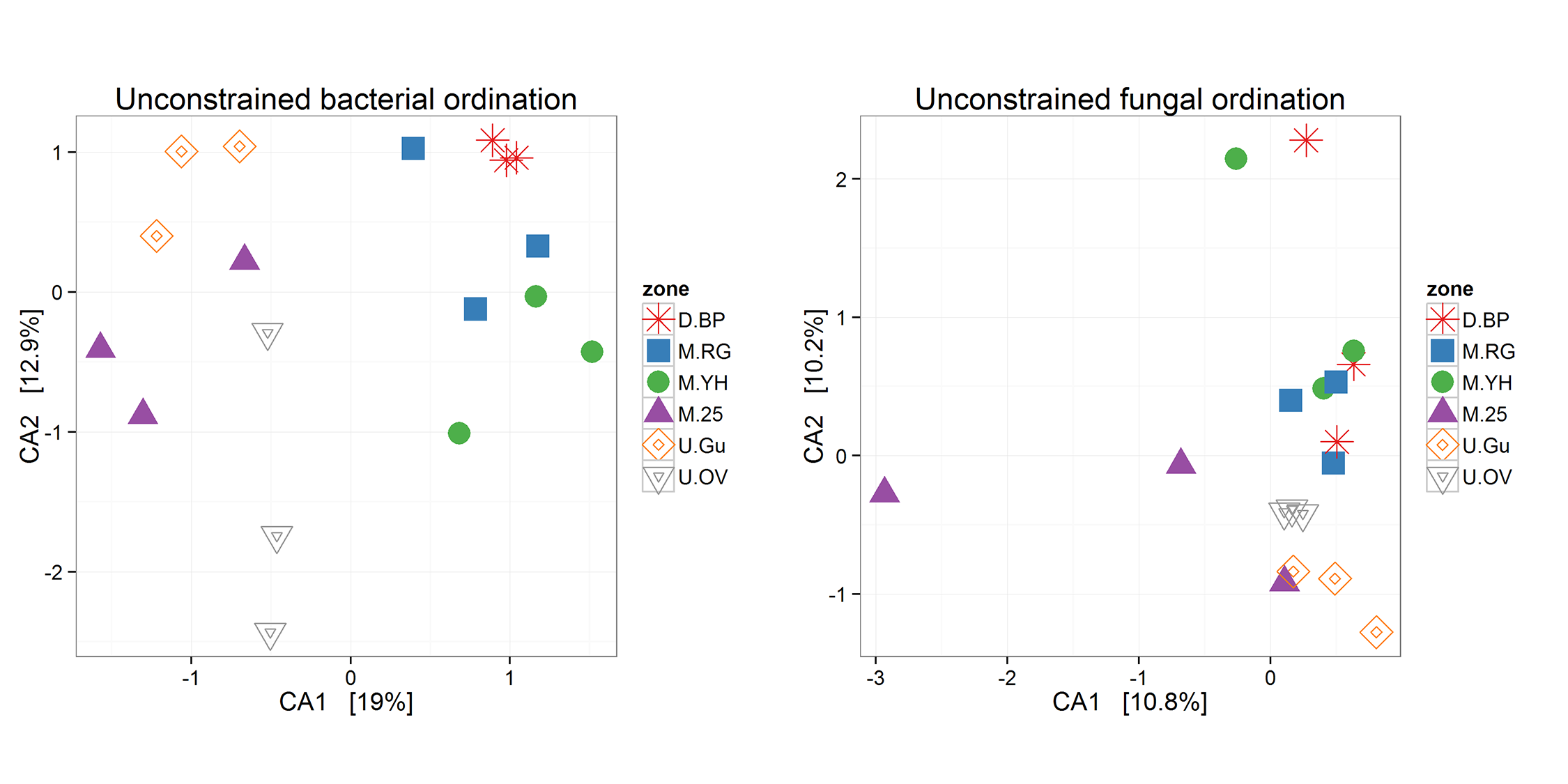

Supplement: S1 Fig — Analysis is based on relative abundance of 354 bacterial OTUS and 273 fungal OTUs across six zone classifications (Table 1). Markers indicate individual samples (three per zone type). Scree plots are provided in S2 Fig. (TIF) [file pone.0124726.s006.tif]

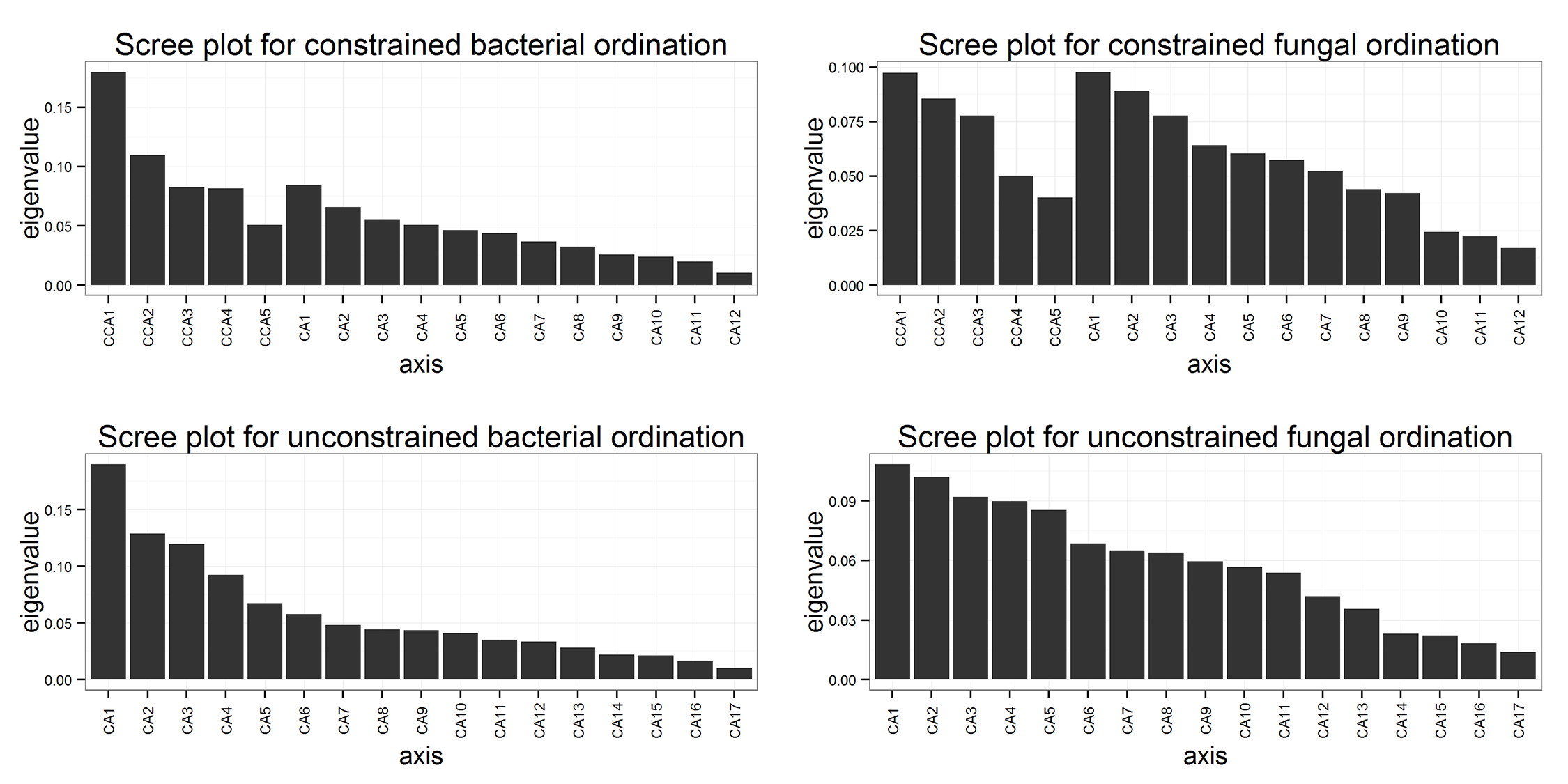

Supplement: S2 Fig — (TIF) [file pone.0124726.s007.tif]

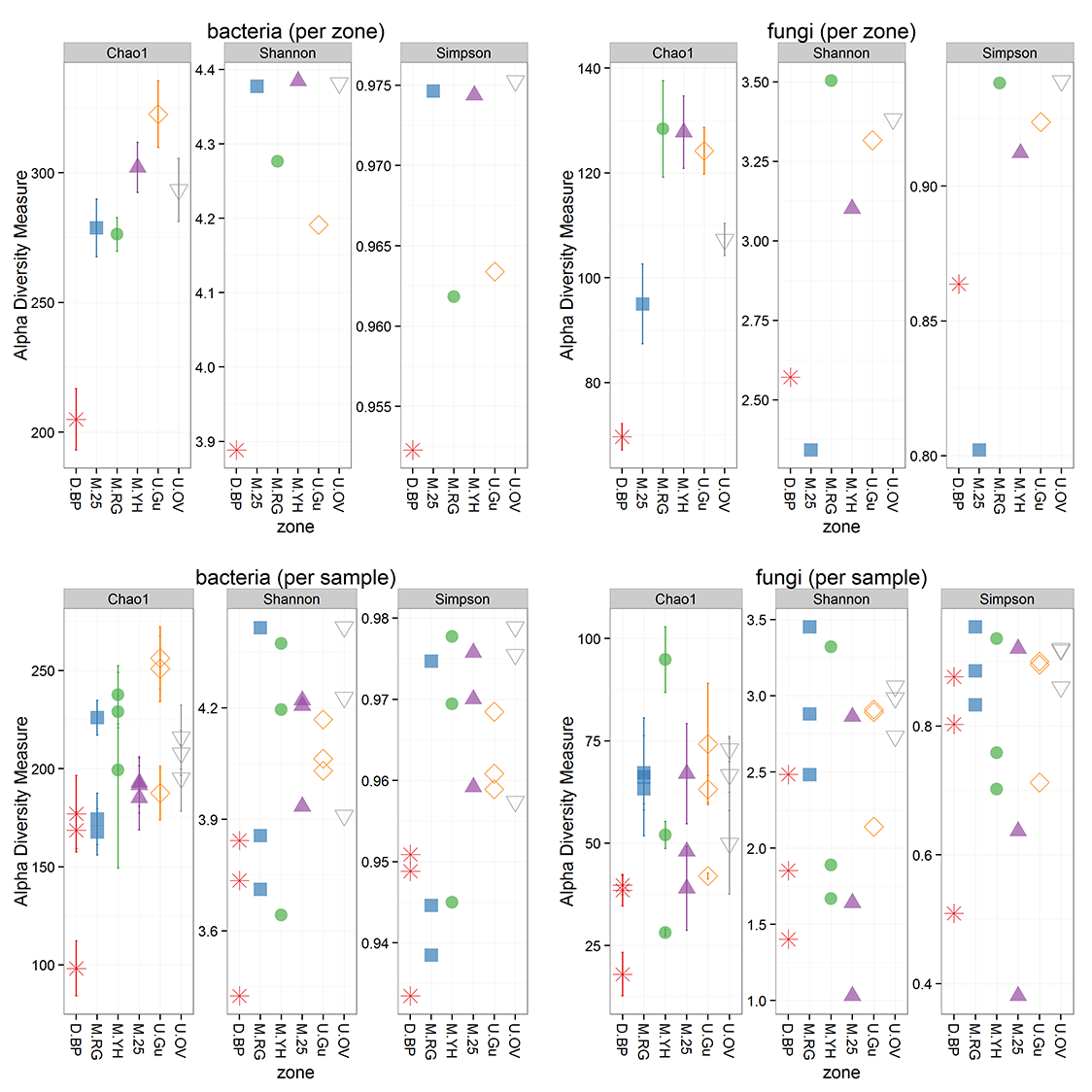

Supplement: S3 Fig — Based on 3 samples per zone analysed separately (mean 1742 sequences), and together (mean 5460 sequences). (TIF) [file pone.0124726.s008.tif]

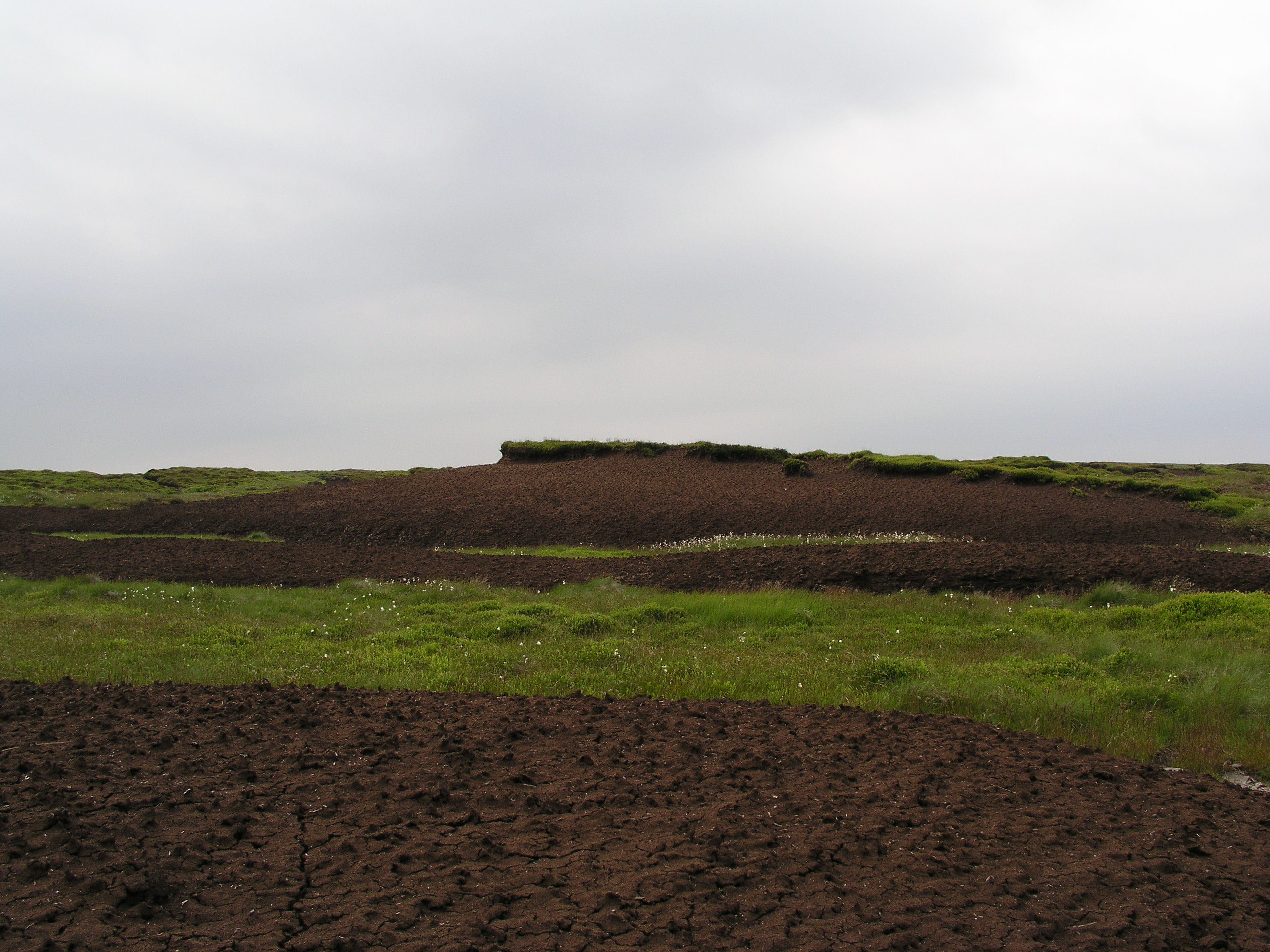

Supplement: S4 Fig — Note bare unconsolidated peat areas, cotton grass and dwarf shrub dominated gully vegetation and elevated remnants of the peat dome supporting original dwarf shrub vegetation. Photograph taken July 2006. (JPG) [file pone.0124726.s009.jpg]

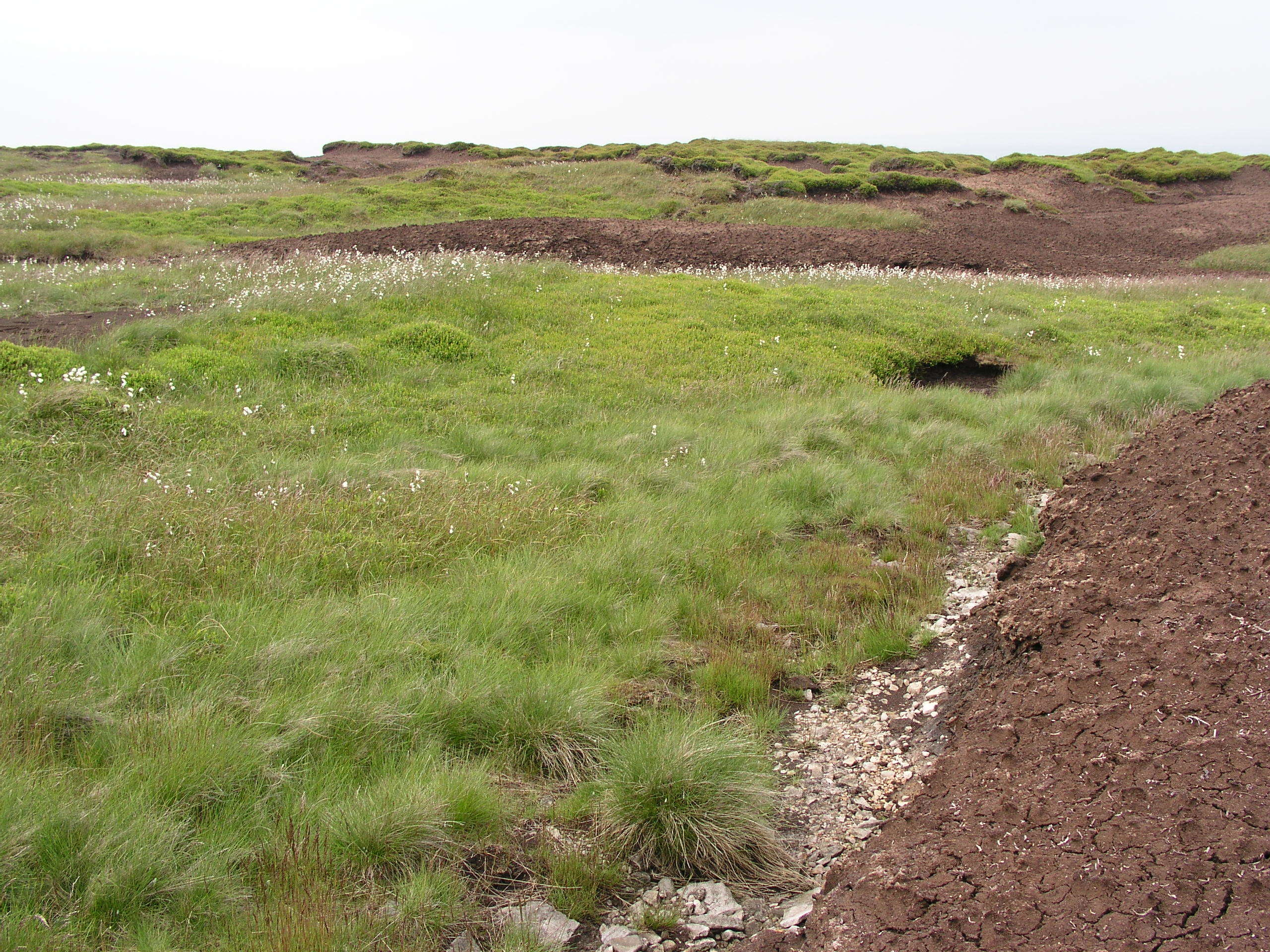

Supplement: S5 Fig — Note bare unconsolidated peat gully walls and exposed gritstone bedrock margins in a gully supporting naturally regenerated cotton grass/grass/dwarf shrub dominated vegetation and elevated remnants of the peat dome supporting original dwarf shrub vegetation in the background. Photograph taken July 2006. (JPG) [file pone.0124726.s010.jpg]
