# Supplementary material for: Bacterial and Fungal Communities in a Degraded Ombrotrophic Peatland Undergoing Natural and Managed Re-Vegetation
Source: PLoS One. 2015 May 13;10(5):e0124726. doi: 10.1371/journal.pone.0124726 (PMC4430338; doi:10.1371/journal.pone.0124726)
Supplement: S1 Table — Sample locations, physico-chemical data, and cultivable microbes from bare peat and the five vegetated zones (see Table 1) at Holme Moss. (DOCX) [file pone.0124726.s002.docx]

| **#SampleID** | **Transect** | **management** | **zone** | **altitude** | **X** | **Y** | **Pb** | **Cu** | **Zn** | **Cd** | **K** | **P** | **Nitrate** | **Ammonium** | **OM** | **Moisture** | **pH** | **Conductivity** | **cfu_per_g_bacteria** | **cfu_per_g_fungi** |
| --- | --- | --- | --- | --- | --- | --- | --- | --- | --- | --- | --- | --- | --- | --- | --- | --- | --- | --- | --- | --- |
| W1 | West | Managed | M.25 | 539.6616 | -1.86011 | 53.53459 | 36.33781 | 86.19331 | 181.0148 | 1.761983 | 1071.038 | 413.4608 | 0.557362 | 4.329741 | 55.30333 | 65.4 | 4.106667 | 37.21 | 745000 | 160000 |
| W2 | West | Degraded | D.BP | 538.2197 | -1.86019 | 53.53603 | 4.670806 | 3.184644 | 60.46105 | 0.46 | 133.653 | 296.9303 | 18.08069 | 7.796736 | 93.56333 | 84.39667 | 3.81 | 64.43333 | 4800 | 4500 |
| W3 | West | Managed | M.YH | 536.5375 | -1.86015 | 53.53646 | 20.83976 | 9.38269 | 219.1123 | 1.366491 | 467.8423 | 384.7822 | 2.038312 | 5.164387 | 94.36 | 81.65333 | 3.656667 | 67.16667 | 370000 | 275000 |
| W4 | West | Unmanaged | U.Gu | 535.5761 | -1.86009 | 53.53675 | 43.71505 | 15.68762 | 121.3469 | 1.168889 | 383.8369 | 587.4624 | 23.96953 | 10.71134 | 77.11333 | 63.97 | 3.936667 | 61.16667 | 4500000 | 1000000 |
| W5 | West | Managed | M.RG | 536.5375 | -1.8602 | 53.53677 | 5.069785 | 12.15457 | 66.31911 | 0.388039 | 367.8458 | 364.4677 | 0.569778 | 4.737864 | 97.94 | 86.34 | 3.913333 | 51.06667 | 2010000 | 705000 |
| W6 | West | Unmanaged | U.OV | 538.7003 | -1.86015 | 53.53689 | 453.9285 | 59.23095 | 194.091 | 2.516536 | 3041.992 | 943.214 | 17.40298 | 24.9987 | 85.80333 | 67.93 | 3.926667 | 71.36667 | 1505000 | 740000 |
| M1 | Middle | Managed | M.25 | 536.0568 | -1.85868 | 53.5347 | 126.0542 | 24.88722 | 239.548 | 2.079 | 424.9093 | 475.0133 | 0.199907 | 9.537073 | 91.44333 | 75.57667 | 3.893333 | 63.63333 | 10100000 | 5700000 |
| M2 | Middle | Degraded | D.BP | 532.4519 | -1.8586 | 53.53642 | 1.925549 | 4.640859 | 84.53996 | 0.514626 | 118.7192 | 251.616 | 12.52934 | 5.404594 | 98.18667 | 83.10667 | 3.85 | 83.93333 | 2600 | 3800 |
| M3 | Middle | Managed | M.YH | 531.4906 | -1.8586 | 53.53676 | 2.98248 | 4.428751 | 57.06114 | 0.365167 | 385.1442 | 326.5282 | 0.556875 | 5.500755 | 97.93 | 84.03667 | 3.873333 | 72.36667 | 500000 | 135000 |
| M4 | Middle | Unmanaged | U.OV | 532.6923 | -1.85867 | 53.53697 | 137.5342 | 24.56736 | 156.5363 | 1.659415 | 423.6955 | 668.7346 | 6.270406 | 17.78308 | 92.77 | 64.74667 | 3.636667 | 92.83333 | 5900000 | 740000 |
| M5 | Middle | Unmanaged | U.Gu | 528.847 | -1.8587 | 53.53736 | 24.79987 | 12.10004 | 111.9843 | 0.788693 | 413.333 | 480.7446 | 6.283135 | 13.62254 | 73.87667 | 65.04333 | 3.926667 | 53.26667 | 10050000 | 1700000 |
| M6 | Middle | Managed | M.RG | 531.2502 | -1.85862 | 53.53761 | 3.459471 | 6.768422 | 42.29183 | 0.594333 | 444.7737 | 347.341 | 0.335718 | 5.180444 | 97.05333 | 82.32333 | 4.063333 | 48.06667 | 1810000 | 1130000 |
| E1 | East | Managed | M.25 | 532.9325 | -1.85774 | 53.53481 | 10.90162 | 10.58291 | 132.2411 | 0.710163 | 686.8802 | 266.9594 | 0.403829 | 2.532635 | 31.22333 | 43.97 | 4.423333 | 50.83333 | 3850000 | 3400000 |
| E2 | East | Managed | M.RG | 530.0486 | -1.85778 | 53.53553 | 1.963834 | 5.491896 | 17.43048 | 0.548823 | 290.8181 | 316.8273 | 0.482616 | 5.862072 | 98.34667 | 85.66667 | 3.926667 | 54.5 | 1195000 | 470000 |
| E3 | East | Degraded | D.BP | 527.405 | -1.85781 | 53.53663 | 3.898973 | 6.000732 | 118.0507 | 0.687623 | 275.8518 | 321.7897 | 12.39749 | 9.306487 | 98.41667 | 84.76333 | 3.746667 | 67.7 | 108500 | 73000 |
| E4 | East | Managed | M.YH | 526.2034 | -1.85785 | 53.53715 | 6.489661 | 9.995274 | 169.9949 | 1.379667 | 860.4787 | 367.9937 | 0.426338 | 6.961193 | 98.02667 | 85.02333 | 3.71 | 58.73333 | 1765000 | 610000 |
| E5 | East | Unmanaged | U.Gu | 523.0791 | -1.85775 | 53.53743 | 100.7468 | 24.93053 | 70.6314 | 1.600667 | 681.3097 | 912.4437 | 54.59817 | 24.07183 | 82.21 | 76.52667 | 3.766667 | 74.8 | 4150000 | 750000 |
| E6 | East | Unmanaged | U.OV | 526.9244 | -1.85777 | 53.5375 | 305.2813 | 48.17148 | 111.2516 | 2.897269 | 636.8033 | 762.8847 | 12.8167 | 16.62546 | 86.55 | 67.80333 | 3.633333 | 100.7667 | 3350000 | 500000 |
