# Supplementary material for: Bacterial and Fungal Communities in a Degraded Ombrotrophic Peatland Undergoing Natural and Managed Re-Vegetation
Source: PLoS One. 2015 May 13;10(5):e0124726. doi: 10.1371/journal.pone.0124726 (PMC4430338; doi:10.1371/journal.pone.0124726)
Supplement: S2 Table — Relative abundances are expressed as a percentage within each kingdom (i.e. columns add up to 200%). (DOCX) [file pone.0124726.s003.docx]

| **Kingdom** | **Phylum** | **D.BP** | **M.25** | **M.RG** | **M.YH** | **U.Gu** | **U.OV** |
| --- | --- | --- | --- | --- | --- | --- | --- |
| Bacteria | Acidobacteria | 33.92098 | 38.5712 | 19.80353 | 19.82141 | 43.4922 | 28.71728 |
| Bacteria | Proteobacteria | 49.57481 | 49.93014 | 55.48543 | 48.95032 | 44.88278 | 51.31611 |
| Bacteria | Bacteroidetes | 0.791689 | 3.317881 | 2.8915 | 2.224229 | 2.723133 | 4.486187 |
| Bacteria | Firmicutes | 0.027801 | 0.090765 | 0 | 0 | 0 | 0.061275 |
| Bacteria | OD1 | 0 | 0.045382 | 0.051177 | 0 | 0 | 0 |
| Bacteria | Actinobacteria | 9.472532 | 5.548596 | 11.62707 | 12.04418 | 4.695691 | 10.81883 |
| Bacteria | TM7 | 0 | 0.112237 | 0.104445 | 0.083752 | 0.071292 | 0.288751 |
| Bacteria | Spirochaetes | 0 | 0.293461 | 0.017059 | 0.029855 | 0.09143 | 0 |
| Bacteria | AD3 | 2.913566 | 0.224289 | 5.621563 | 11.43326 | 1.526174 | 2.286643 |
| Bacteria | Verrucomicrobia | 1.166708 | 0.729752 | 1.815798 | 3.255455 | 0.959511 | 0.429923 |
| Bacteria | WPS-2 | 0.653946 | 0.240581 | 0.20889 | 0.294419 | 0.201686 | 0.465714 |
| Bacteria | TM6 | 0.700183 | 0.251572 | 1.274348 | 0.919193 | 0.352948 | 0.129413 |
| Bacteria | Chlorobi | 0.23576 | 0.032584 | 0.262158 | 0.038256 | 0.550232 | 0.316597 |
| Bacteria | FCPU426 | 0.274352 | 0 | 0.302935 | 0.092406 | 0.061736 | 0.030637 |
| Bacteria | Armatimonadetes | 0.042415 | 0.087554 | 0.068236 | 0.167951 | 0.07997 | 0.180607 |
| Bacteria | Elusimicrobia | 0.196747 | 0.491424 | 0.297851 | 0.482011 | 0.11996 | 0.038895 |
| Bacteria | Cyanobacteria | 0.028514 | 0.032584 | 0 | 0.040129 | 0.030868 | 0.016088 |
| Bacteria | Gemmatimonadetes | 0 | 0 | 0 | 0.053183 | 0.04145 | 0 |
| Bacteria | OP3 | 0 | 0 | 0 | 0.069984 | 0.029842 | 0.097238 |
| Bacteria | Tenericutes | 0 | 0 | 0 | 0 | 0.089092 | 0.319817 |
| Bacteria | Chloroflexi | 0 | 0 | 0.168011 | 0 | 0 | 0 |
| Fungi | Ascomycota | 53.17274 | 77.72568 | 81.4575 | 59.3594 | 64.51566 | 55.7519 |
| Fungi | Glomeromycota | 0.809637 | 0 | 0.092081 | 1.117147 | 0.402877 | 0.111782 |
| Fungi | Zygomycota | 1.133155 | 1.317137 | 0.932831 | 0.835071 | 3.767433 | 22.0978 |
| Fungi | Chytridiomycota | 0.203817 | 0.176056 | 0 | 0.015768 | 0.15441 | 0.228258 |
| Fungi | Basidiomycota | 44.68065 | 20.78113 | 17.51759 | 38.67261 | 31.15962 | 21.81026 |
