# Supplementary material for: Bacterial and Fungal Communities in a Degraded Ombrotrophic Peatland Undergoing Natural and Managed Re-Vegetation
Source: PLoS One. 2015 May 13;10(5):e0124726. doi: 10.1371/journal.pone.0124726 (PMC4430338; doi:10.1371/journal.pone.0124726)
Supplement: S3 Table — (DOCX) [file pone.0124726.s004.docx]

| **Phylum** | **D.BP** | **U.OV** | **D.BP.expected** | **U.OV.expected** | **Xsq_p.value** | **Xsq_p.value.corrected** | **D.BP_pc** | **U.OV_pc** | **diff** |
| --- | --- | --- | --- | --- | --- | --- | --- | --- | --- |
| Zygomycota | 58 | 557 | 351.0458 | 263.9542 | 0 | 0 | 1.383588 | 17.67132 | -16.2877 |
| Ascomycota | 2121 | 1882 | 2284.937 | 1718.063 | 1.65E-07 | 2.20E-07 | 50.59637 | 59.70812 | -9.11175 |
| Bacteroidetes | 27 | 204 | 103.2607 | 127.7393 | 0 | 0 | 0.685279 | 4.185474 | -3.50019 |
| Actinobacteria | 380 | 546 | 413.9369 | 512.0631 | 0.02489 | 0.039824 | 9.64467 | 11.2023 | -1.55763 |
| Proteobacteria | 1980 | 2483 | 1995.033 | 2467.967 | 0.650839 | 0.650839 | 50.25381 | 50.94378 | -0.68998 |
| WPS-2 | 29 | 27 | 25.0329 | 30.9671 | 0.286308 | 0.32721 | 0.736041 | 0.55396 | 0.182081 |
| TM6 | 29 | 7 | 16.09258 | 19.90742 | 1.51E-05 | 6.05E-05 | 0.736041 | 0.143619 | 0.592421 |
| AD3 | 115 | 112 | 101.4727 | 125.5273 | 0.070942 | 0.094589 | 2.918782 | 2.297907 | 0.620874 |
| Verrucomicrobia | 44 | 19 | 28.16201 | 34.83799 | 5.99E-05 | 0.00016 | 1.116751 | 0.389824 | 0.726928 |
| Glomeromycota | 41 | 5 | 26.25708 | 19.74292 | 1.12E-05 | 1.12E-05 | 0.978053 | 0.158629 | 0.819424 |
| Acidobacteria | 1308 | 1409 | 1214.543 | 1502.457 | 0.000311 | 0.000621 | 33.19797 | 28.90849 | 4.289475 |
| Basidiomycota | 1961 | 705 | 1521.769 | 1144.231 | 0 | 0 | 46.77958 | 22.36675 | 24.41283 |
