# Supplementary material for: Bacterial and Fungal Communities in a Degraded Ombrotrophic Peatland Undergoing Natural and Managed Re-Vegetation
Source: PLoS One. 2015 May 13;10(5):e0124726. doi: 10.1371/journal.pone.0124726 (PMC4430338; doi:10.1371/journal.pone.0124726)
Supplement: S4 Table — (DOCX) [file pone.0124726.s005.docx]

| **variable** | **factor** | **W** | **P** |
| --- | --- | --- | --- |
| pH | bare peat and early stage restoration/other | 31 | 0.42582 |
| Moisture | bare peat and early stage restoration/other | 81 | 0.00004 |
| OM | bare peat and early stage restoration/other | 81 | 0.00004 |
| Ammonium | bare peat and early stage restoration/other | 18 | 0.05031 |
| Nitrate | bare peat and early stage restoration/other | 30 | 0.38651 |
| P | bare peat and early stage restoration/other | 8 | 0.00276 |
| K | bare peat and early stage restoration/other | 16 | 0.03147 |
| Pb | bare peat and early stage restoration/other | 1 | 0.00008 |
| Cd | bare peat and early stage restoration/other | 6 | 0.00123 |
| Cu | bare peat and early stage restoration/other | 2 | 0.00016 |
| bacteria_cfu | bare peat and early stage restoration/other | 7 | 0.00185 |
| fungi_cfu | bare peat and early stage restoration/other | 12 | 0.01337 |
| pH | Original vegetation (U.OV)/other | 35 | 0.15428 |
| Moisture | Original vegetation (U.OV)/other | 35 | 0.16422 |
| OM | Original vegetation (U.OV)/other | 29 | 0.49755 |
| Ammonium | Original vegetation (U.OV)/other | 2 | 0.0098 |
| Nitrate | Original vegetation (U.OV)/other | 12 | 0.25 |
| P | Original vegetation (U.OV)/other | 2 | 0.0098 |
| K | Original vegetation (U.OV)/other | 11 | 0.20343 |
| Pb | Original vegetation (U.OV)/other | 0 | 0.00245 |
| Cd | Original vegetation (U.OV)/other | 2 | 0.0098 |
| Cu | Original vegetation (U.OV)/other | 5 | 0.03922 |
| bacteria_cfu | Original vegetation (U.OV)/other | 15 | 0.42647 |
| fungi_cfu | Original vegetation (U.OV)/other | 20 | 0.81261 |
| pH | Bare peat (D.BP)/other | 31 | 0.34225 |
| Moisture | Bare peat (D.BP)/other | 10 | 0.16422 |
| OM | Bare peat (D.BP)/other | 7 | 0.07598 |
| Ammonium | Bare peat (D.BP)/other | 24 | 0.91176 |
| Nitrate | Bare peat (D.BP)/other | 10 | 0.16422 |
| P | Bare peat (D.BP)/other | 42 | 0.01716 |
| K | Bare peat (D.BP)/other | 45 | 0.00245 |
| Pb | Bare peat (D.BP)/other | 39 | 0.05637 |
| Cd | Bare peat (D.BP)/other | 37 | 0.10049 |
| Cu | Bare peat (D.BP)/other | 42 | 0.01716 |
| bacteria_cfu | Bare peat (D.BP)/other | 45 | 0.00245 |
| fungi_cfu | Bare peat (D.BP)/other | 45 | 0.00912 |
| pH | Managed/Unmanaged including bare peat | 56 | 0.18442 |
| Moisture | Managed/Unmanaged including bare peat | 54 | 0.25808 |
| OM | Managed/Unmanaged including bare peat | 48 | 0.5457 |
| Ammonium | Managed/Unmanaged including bare peat | 6 | 0.00123 |
| Nitrate | Managed/Unmanaged including bare peat | 0 | 0.00004 |
| P | Managed/Unmanaged including bare peat | 24 | 0.1615 |
| K | Managed/Unmanaged including bare peat | 52 | 0.34011 |
| Pb | Managed/Unmanaged including bare peat | 25 | 0.19025 |
| Cd | Managed/Unmanaged including bare peat | 31 | 0.43628 |
| Cu | Managed/Unmanaged including bare peat | 34 | 0.60481 |
| bacteria_cfu | Managed/Unmanaged including bare peat | 38 | 0.86331 |
| fungi_cfu | Managed/Unmanaged including bare peat | 46 | 0.65868 |
